# Manganese-catalyzed C–C and C–N bond formation with alcohols via borrowing hydrogen or hydrogen auto-transfer

**DOI:** 10.3762/bjoc.20.98

**Published:** 2024-05-21

**Authors:** Mohd Farhan Ansari, Atul Kumar Maurya, Abhishek Kumar, Saravanakumar Elangovan

**Affiliations:** 1 Department of Chemistry, Indian Institute of Technology (BHU), Varanasi, Uttar Pradesh 221005, India

**Keywords:** alcohols, alkylation, amines, borrowing hydrogen, hydrogen auto-transfer, manganese

## Abstract

Transition-metal-mediated “borrowing hydrogen" also known as hydrogen auto-transfer reactions allow the sustainable construction of C–C and C–N bonds using alcohols as hydrogen donors. In recent years, manganese complexes have been explored as efficient catalysts in these reactions. This review highlights the significant progress made in manganese-catalyzed C–C and C–N bond-formation reactions via hydrogen auto-transfer, emphasizing the importance of this methodology and manganese catalysts in sustainable synthesis strategies.

## Introduction

**Scheme 1:**
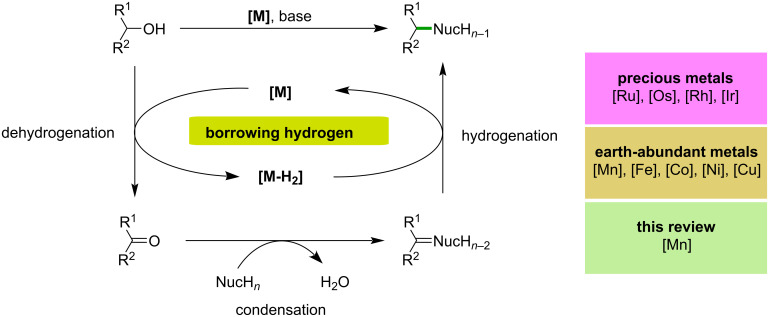
General scheme of the borrowing hydrogen (BH) or hydrogen auto-transfer (HA) methodology.

The construction of C–C and C–N bonds is of utmost importance in organic synthesis and is widely used in the pharmaceutical and other chemical industries. Palladium-catalyzed cross-coupling reactions are one of the compelling methods for building C–C and C–N bonds [1–2]. However, using organohalide reagents and harsh reaction conditions in this process results in the co-production of a significant amount of waste or side-products. Borrowing hydrogen (BH) or hydrogen auto-transfer (HA) reactions have emerged as the most elegant and powerful strategy to overcome this drawback [3–5]. Furthermore, this method has been considered an environmentally friendly and atom-economical process for C–C and C–N bond formations utilizing alcohol as an alkylating agent and hydrogen donor, producing water as the only side-product [6–9]. Notably, alcohols are inexpensive, abundant and can be obtained from biomass, which makes this method even more attractive to the scientific community [10–12]. In this process, first, the metal-catalyzed dehydrogenation of the alcohol provides a reactive substrate for coupling with nucleophiles and the active metal hydride species. Later, the borrowed hydrogen is used in the final step to reduce unsaturated compounds. To achieve the selective C–C and C–N bond formation via hydrogen borrowing, controlling the selectivity is an important factor since the formation of possible side-products such as overreduction of unsaturated compounds or dialkylation. Hence, developing an efficient catalyst, capable of achieving both selective dehydrogenation and hydrogenation is highly important. A typical BH process is demonstrated in Scheme 1.

Several precious transition-metal catalysts have been used successfully in this area, including iridium, rhodium, ruthenium, and osmium [4]. However, these noble metals are toxic, expensive, and limited in availability. Hence, replacing them with the first row of transition metals would increase the sustainability and profitability of this procedure [13]. Indeed, many 3d-metal-based homogeneous catalysts have been documented for BH reactions [14–15] since these metals are considerably inexpensive, eco-friendly and more abundant in the Earth’s crust. According to this viewpoint, manganese is biocompatible and less expensive than noble metals. Also, it is the third most abundant transition metal, behind titanium and iron. After the independent pioneering works of Beller [16] and Milstein [17] in hydrogenation and dehydrogenation reactions with pincer-decorated manganese complexes, significant progress has been made in manganese catalysis [18–20]. Notably, well-defined low-valent diamagnetic manganese(I) complexes have been studied in many catalytic transformations, and several reviews have been reported on their applications in dehydrogenative coupling reactions [21–24]. This review focuses mainly on the BH reaction to create sustainable C–C and C–N bonds with manganese catalysts.

## Review

### C–N bond formation with alcohols and amines

**Scheme 2:**
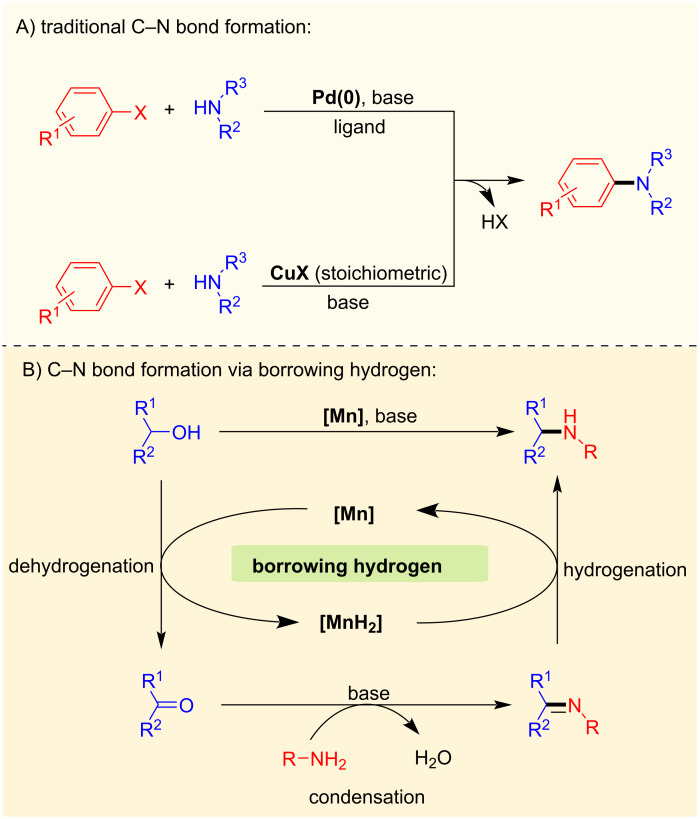
General scheme for C–N bond formation. A) Traditional cross-couplings with alkyl or aryl halides. B) Borrowing hydrogen with alcohols and amines.

Amines and their derivatives are of substantial importance for the fine chemical industry, pharmaceuticals, agrochemicals, dyes, and natural products [25]. The synthesis of amine derivatives can be accomplished using many powerful techniques, including Buchwald–Hartwig and Ullmann cross-coupling reactions, hydroamination, hydroaminomethylation, reduction of nitriles and nitro compounds or through reductive amination of carbonyl derivatives [26–30]. However, for example, cross-coupling reactions with alkyl or aryl halides generate considerable amounts of waste (Scheme 2A). Even though many different approaches exist for synthesizing amines, the borrowing hydrogen approach is becoming increasingly popular in catalysis since this method provides an excellent example of a green chemistry and atom-efficient reaction [31–33]. This section focuses on manganese-catalyzed C–N bond formation reactions via BH or HA using alcohols as hydrogen donors and alkylating agents.

**Figure 1:**
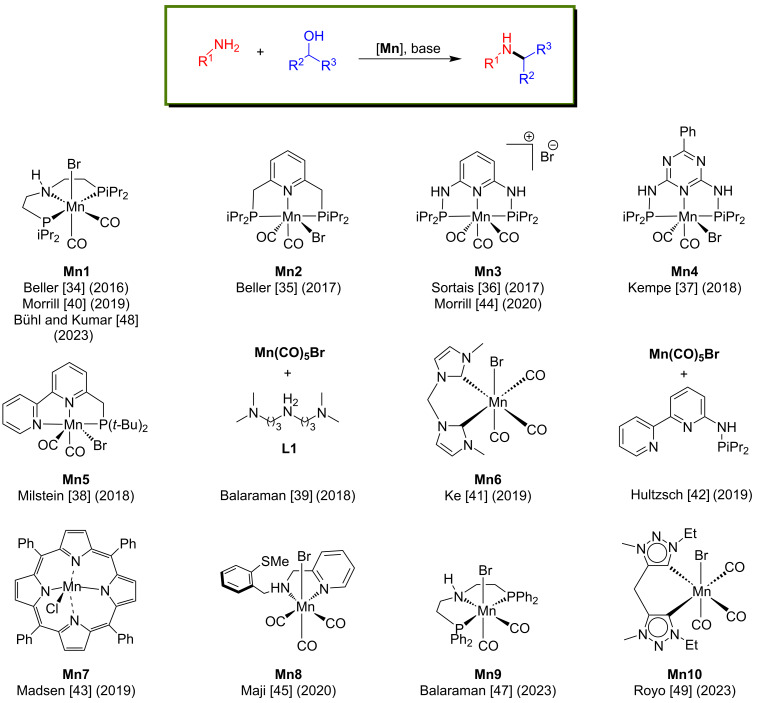
Manganese pre-catalysts used for the N-alkylation of amines with alcohols.

In general, low-valent manganese complexes are used as pre-catalysts in this reaction and are activated using a strong base to generate the active amido complexes, which in turn activate the alcohols. Then, the formed dehydrogenation products, such as aldehydes or ketones, undergo base-assisted condensation reactions with amines providing the corresponding imines. In the last step, the active manganese hydride complexes reduce the imine compounds and afford the desired alkylated amine products (Scheme 2B). Several well-defined manganese complexes have been developed for the N-alkylation of amines with alcohols, including methanol (Figure 1).

**Scheme 3:**
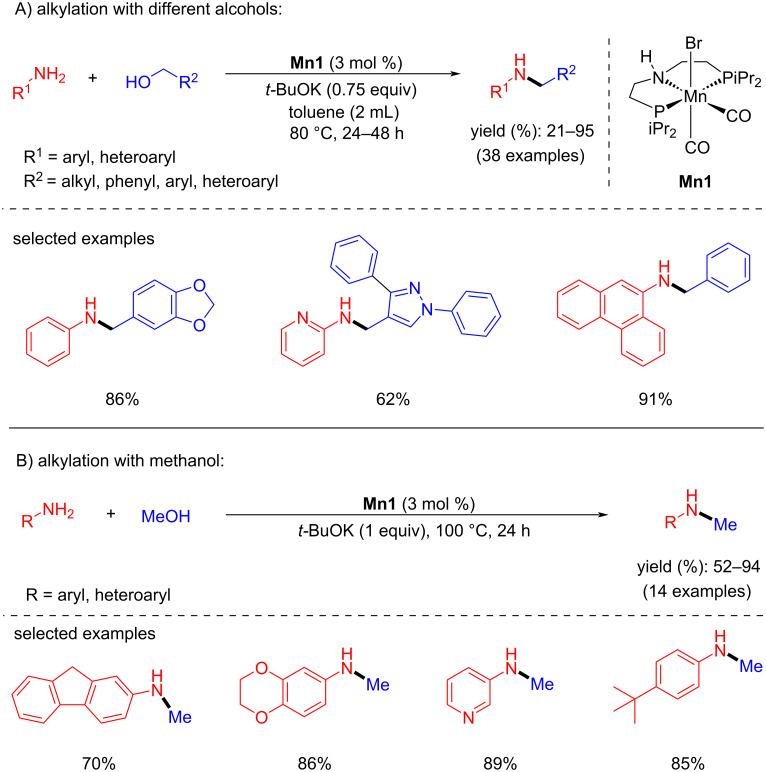
Manganese(I)-pincer complex **Mn1** used for the N-alkylation of amines with alcohols and methanol.

Beller and co-workers introduced the first intriguing manganese-catalyzed BH for the N-alkylation of amines with alcohols in 2016 [34]. The potential Mn(I)-pincer complex **Mn1** (3 mol %) catalyzed the coupling of the several alcohols and primary amines in the presence of *t*-BuOK (0.75 equiv) in toluene at 80 °C for 24–48 h and selectively produced the N-alkylated products with good yields (Scheme 3). More interestingly, the first non-noble-metal catalyzed the most challenging N-methylation of amines with methanol was achieved at 100 °C with one equivalent of *t*-BuOK. In all the cases, the catalytic system selectively yielded mono-N-alkylated and N-methylated products under mild conditions. Noteworthy, high functional group tolerance, such as alkenes, halogens, thioethers, and benzodioxane derivatives was observed under the established reaction conditions. This pioneering work opened the door for manganese catalysis in BH reactions. However, the high base loading (0.75–1 equiv) was required for this system to attain good yields of the N-alkylated products.

**Scheme 4:**
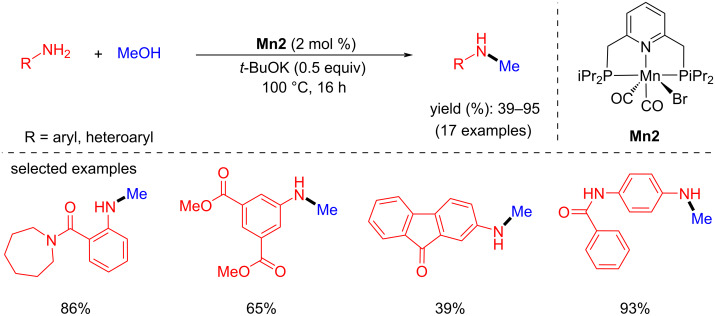
N-Methylation of amines with methanol using **Mn2**.

Later, the same group developed the second generation of manganese PNP pincer complexes for the N-methylation of aromatic amines with methanol [35]. Various primary anilines were methylated selectively with good yields using **Mn2** (2 mol %) and *t*-BuOK (0.5 equiv) as a base at 100 °C for 16 h (Scheme 4). Compared to their previous report, the N-methylation of amines with methanol was achieved with lower catalyst and base loading.

**Scheme 5:**
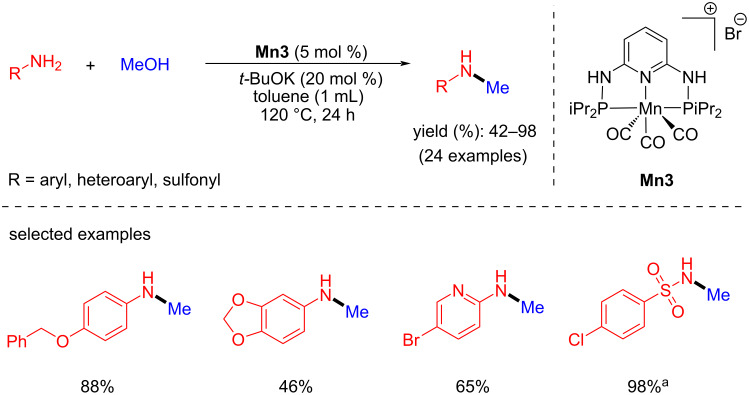
C–N-Bond formation with amines and methanol using PN^3^P-Mn complex **Mn3** reported by Sortais et al. [36]. ^a^1.2 Equiv *t*-BuOK, 60 h.

Sortais et al. reported an elegant example of a manganese-catalyzed N-methylation of primary amines with methanol using catalytic amounts of base. They synthesized a novel Mn(I) complex bearing a bis(diaminopyridine)phosphine ligand (PN^3^P) (**Mn3**) and studied N-methylation reactions in the presence of *t*-BuOK (20 mol %) at 120 °C for 24 h in toluene [36]. This catalytic system tolerated various functional groups, including nitro, ester, amide, and ketones and gave moderate to good yields (42–98%) of the mono-N-methylated products (Scheme 5). Interestingly, the dearomatized intermediate resulting from the reaction of base and **Mn3** was isolated and characterized by X-ray analysis during the mechanistic investigation.

**Scheme 6:**
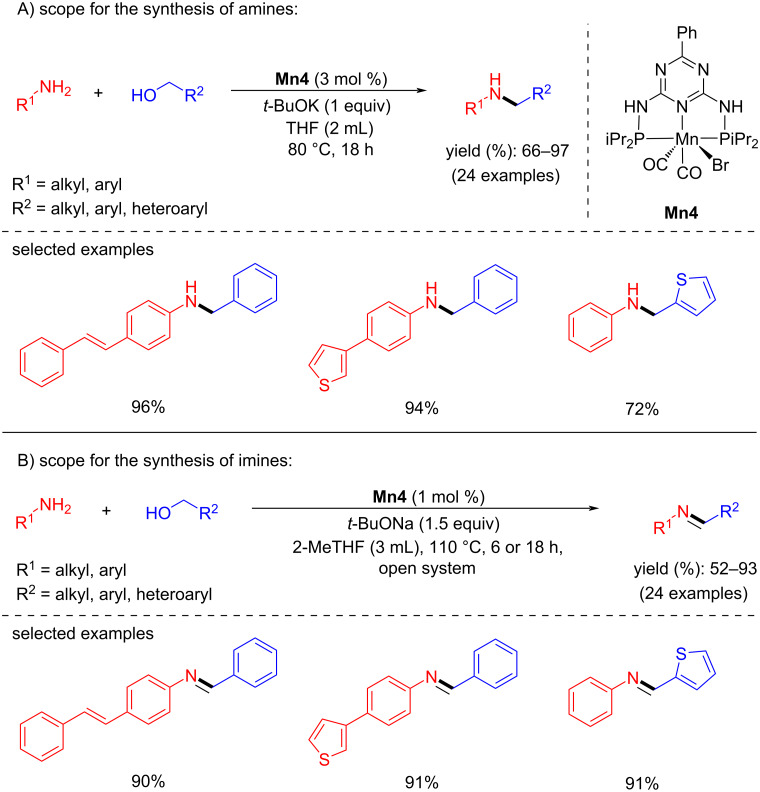
Base-assisted synthesis of amines and imines with **Mn4**. Reaction assisted by A) *t*-BuOK and B) *t*-BuONa as base.

In 2018, Kempe et al. disclosed that the choice of the base plays a critical role in the BH method for the synthesis of amines and imines using Mn-pincer catalyst [37]. When *t*-BuOK (1 equiv) was used as a base, alkylated amine products were observed selectively using alcohol as an alkylating agent, whereas when *t-*BuONa (1.5 equiv) was used as base, alkylated imine products were isolated (Scheme 6). This indicates that the cation-coordinative interaction with the catalyst plays a significant role. Moreover, the mechanistic investigation suggested that the observed selectivity is due to the more reactive potassium manganate hydride towards the hydrogenation of imines to amines than the sodium manganate hydride.

**Scheme 7:**
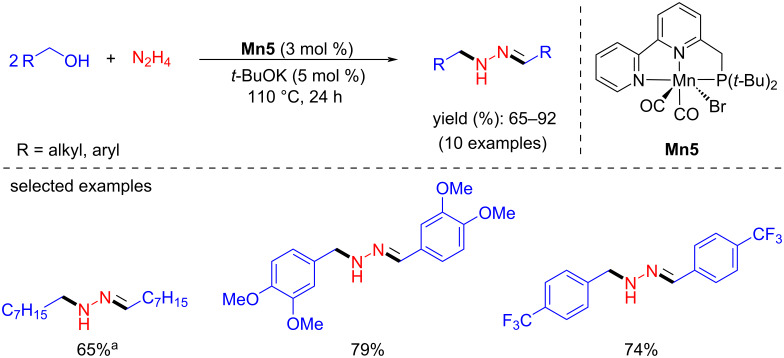
Coupling of alcohols and hydrazine via the HB approach reported by Milstein et al. [38]. ^a^Reaction time was 36 h

In 2018, the Milstein group demonstrated a partial hydrogen-borrowing reaction with a manganese-pincer complex by coupling alcohols and hydrazine to form N-substituted hydrazones. Benzylic and aliphatic alcohols were studied with hydrazine using Mn(*t*-Bu-PNN)(CO)_2_Br (**Mn5**, 3 mol %) and a catalytic amount of *t*-BuOK (5 mol %) at 110 °C [38]. Benzylic alcohols bearing electron-donating and withdrawing groups afforded 65–92% yields of the product within 24 h (Scheme 7). However, aliphatic alcohols such as 1-hexanol and 1-octanol required 36 h to give the corresponding products with 77% and 65% yields, respectively.

**Scheme 8:**
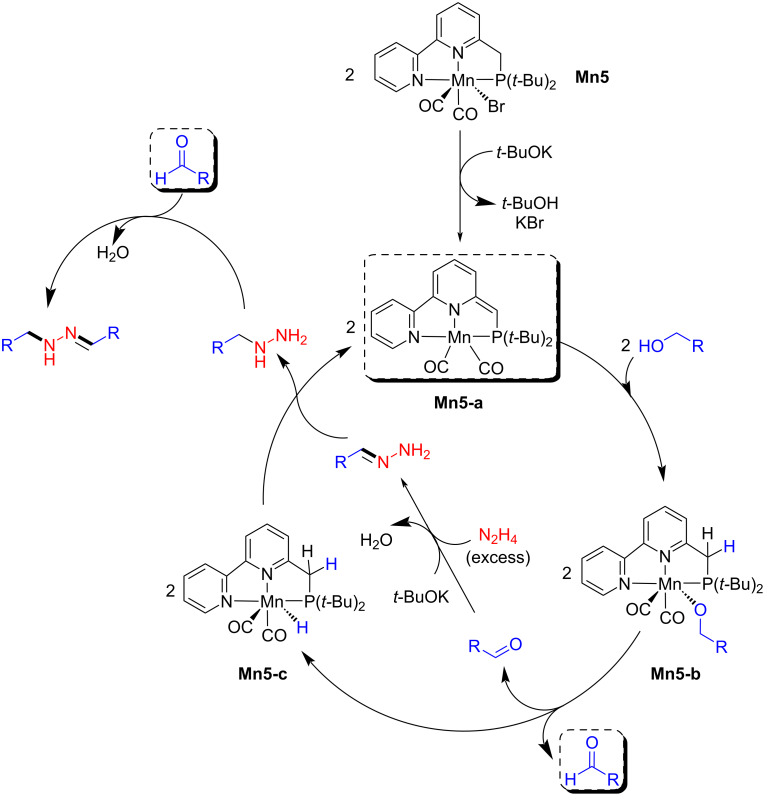
Proposed mechanism for the coupling of alcohols and hydrazine catalyzed by **Mn5**.

The proposed mechanism suggested that the active amido species (**Mn5-a**) was formed by treating **Mn5** with the base. Then, the alkoxy intermediate **Mn5-b** is formed by reaction with the alcohol followed by release of an aldehyde and formation of the manganese hydride **Mn5-c**. The released aldehyde condenses with hydrazine followed by reduction and condensation with another aldehyde to afford the N-substituted hydrazones (Scheme 8).

**Scheme 9:**
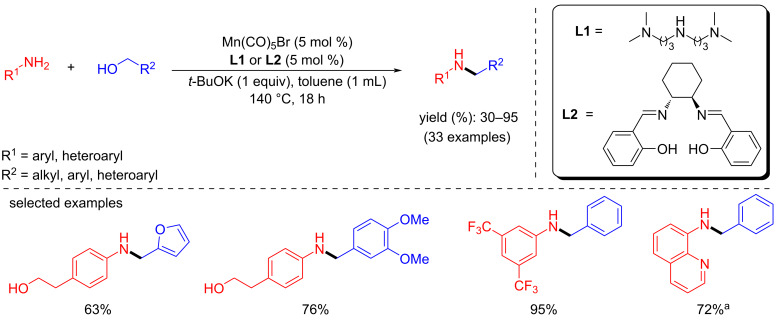
Phosphine-free manganese catalyst for N-alkylation of amines with alcohols reported by Balaraman and co-workers [39]. ^a^Ligand **L2** was used.

Balaraman and co-workers established a phosphine-free manganese catalyst generated in situ from a manganese precursor and a ligand for the N-alkylation of anilines with alcohols [39]. Various ligands were screened for the N-alkylation of *m*-toluidine with benzyl alcohol using Mn(CO)_5_Br (5 mol %) and *t*-BuOK (1 equiv) in toluene at 140 °C (Scheme 9). Among these, **L1** and **L2** showed better activity for the N-alkylation reactions. Different substituted anilines and alcohols, including aliphatic alcohols, were tested and afforded moderate to good yields (up to 95%) of the N-alkylated products using **L1** (5 mol %) and Mn(CO)_5_Br (5 mol %). Notably, heteroaromatic amines provided a good yield with **L2** (5 mol %) under the same reaction conditions. The poisoning test with Hg showed the homogeneous nature of the catalytic system. The mechanistic investigation suggested that the reaction proceeds via a dehydrogenative pathway confirmed by forming an aldehyde product and H_2_ gas which was detected by GC.

**Scheme 10:**
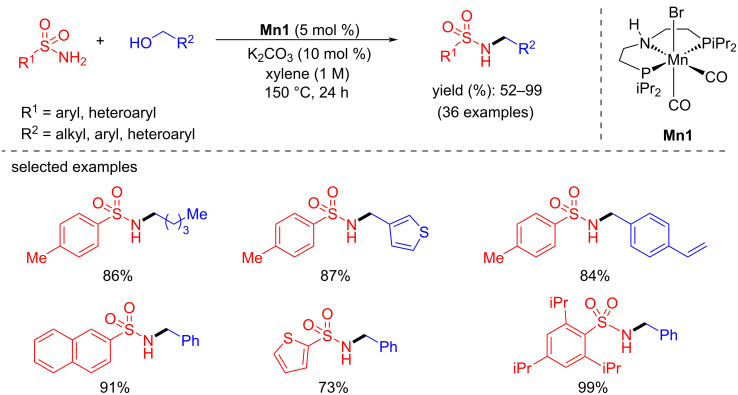
N-Alkylation of sulfonamides with alcohols.

In 2019, Morrill’s group reported the N-alkylation of sulfonamides using **Mn1**. The reaction optimized with 5 mol % of **Mn1** and 10 mol % of K_2_CO_3_ in xylene at high temperature (150 °C) for 24 h afforded the desired N-alkylated sulfonamide compounds [40]. A wide range of aryl and alkyl sulfonamides were alkylated with various benzylic and aliphatic alcohols, providing good to excellent yields (Scheme 10). However, sulfonamides with electron-withdrawing groups attached to the aromatic ring (e.g., 4-NO_2_, 4-CN) were found incompatible with the conditions.

**Scheme 11:**
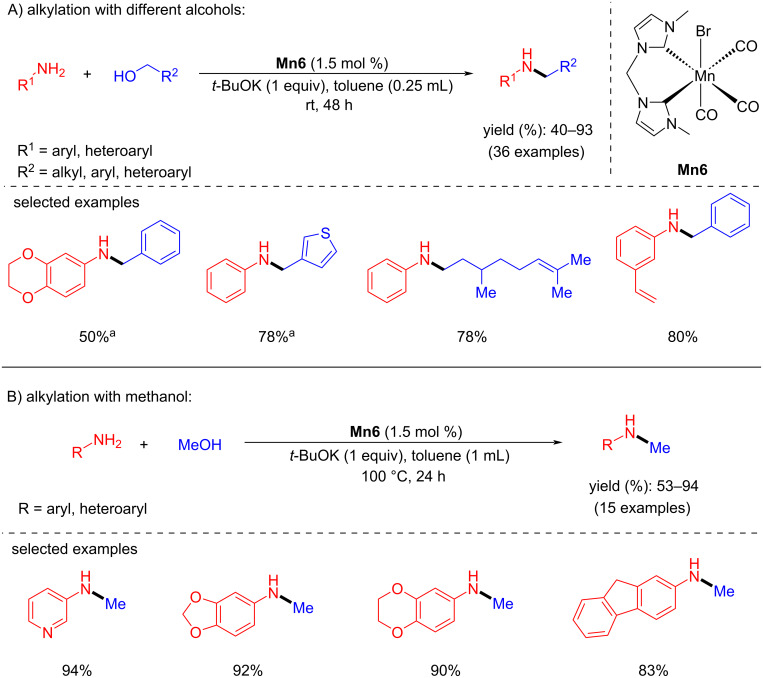
Mn–NHC catalyst **Mn6** applied for the N-alkylation of amines with alcohols. ^a^3 mol % of **Mn6** were used.

Ke and co-workers described an exciting example of a phosphine-free Mn(I)-NHC catalyst for the N-alkylation of amines with alcohols at room temperature [41]. The coupling of several aromatic amines with aliphatic and benzylic alcohols was studied with bis-NHC-manganese complex (**Mn6**). A catalyst loading of 1.5 mol % in the presence of *t*-BuOK (1 equiv) at room temperature produced the corresponding N-alkylated amines with 40–93% yield (Scheme 11). However, N-methylation of anilines with methanol required 100 °C to yield the selective N-methylated products.

**Scheme 12:**
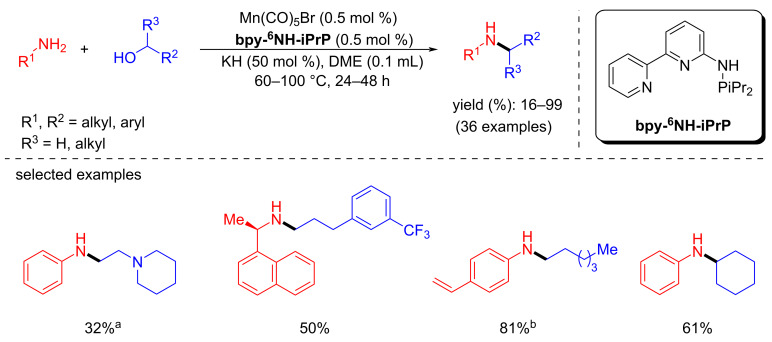
N-Alkylation of amines with primary and secondary alcohols. ^a^80 °C, ^b^100 °C.

The same year, Hultzsch et al. designed PN^3^-pincer ligand-supported Mn(I) complexes for the alkylation of amines with primary and secondary alcohols [42]. Most interestingly, a low catalyst loading (0.5 mol %) and mild reaction conditions (60–100 °C) were employed for this transformation. Aromatic amines gave good yields with benzyl alcohol at 60 °C, but 1,1-phenylethylamine, linear aliphatic amine and benzylamine required 100 °C to achieve the good yields (Scheme 12). Similarly, the N-alkylation of aniline with secondary alcohols required a high temperature (100 °C) compared to substituted benzylic alcohols (60 °C). Interestingly, this protocol was used to synthesize the drug cinacalcet, via alkylating the challenging benzylamine substrate under non-optimized conditions.

**Scheme 13:**
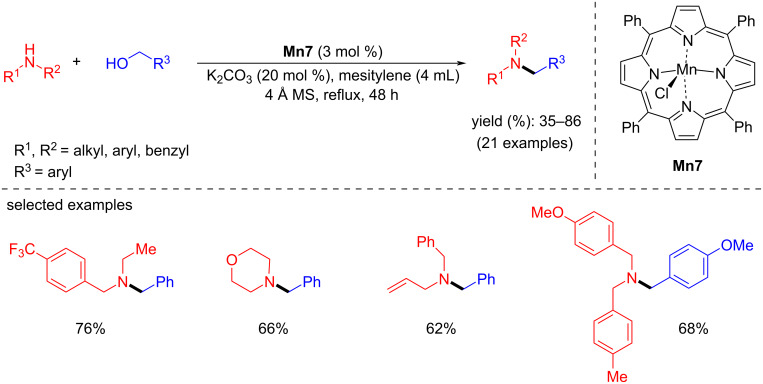
Manganese(III)-porphyrin catalyst for synthesis of tertiary amines.

Later, Madsen’s team introduced a manganese(III) porphyrin system as a catalyst for the BH methodology to achieve C–N coupling reactions [43]. Various tertiary amines were isolated by coupling secondary amines and benzylic alcohols using **Mn7** (3 mol %) in the presence of K_2_CO_3_ (20 mol %) under reflux conditions in mesitylene (Scheme 13).

**Scheme 14:**
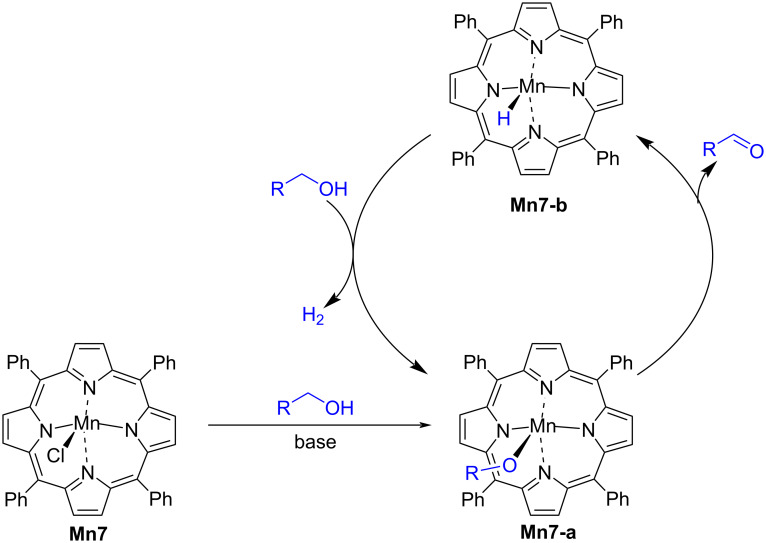
Proposed mechanism for the alcohol dehydrogenation with Mn(III)-porphyrin complex **Mn7**.

The formation of manganese(III) alkoxide intermediate **Mn7-a**, was believed to be the first step in the reaction mechanism which then releases the aldehyde under formation of hydride complex, **Mn7-b**. Then, the alcohol reacts with the hydride complex under release of hydrogen gas and regeneration of complex **Mn7-a** (Scheme 14).

**Scheme 15:**
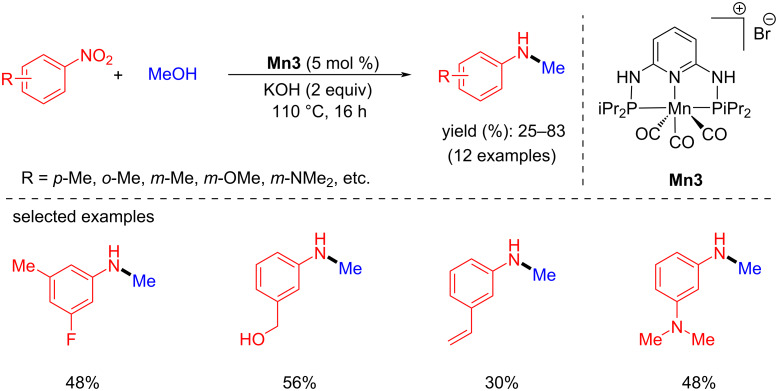
N-Methylation of nitroarenes with methanol using catalyst **Mn3**.

In 2020, Morrill's group reported the one-pot synthesis of *N*-methylarylamines from nitroarenes using methanol as a methylating agent and reductant [44]. When substituted nitroarenes were methylated with methanol under optimal conditions (5 mol % **Mn3**, 2 equiv of KOH at 110 °C for 16 h), moderate to good yields of *N*-methylamines were produced (Scheme 15).

**Scheme 16:**
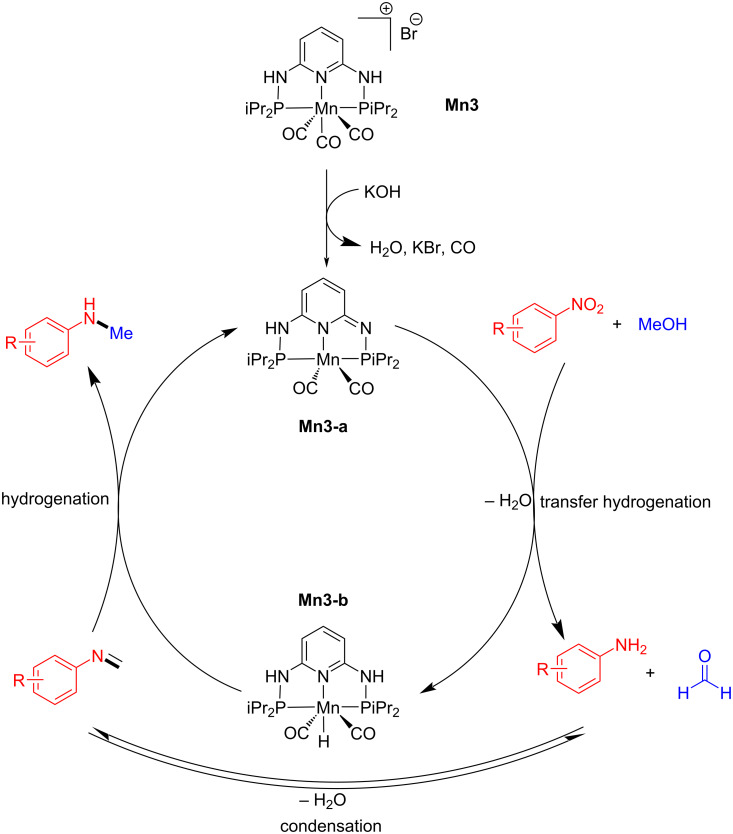
Mechanism of manganese-catalyzed methylation of nitroarenes using **Mn3** as the catalyst.

The mechanistic studies suggested that the base activates the complex **Mn3**. The active catalyst dehydrogenates methanol into formaldehyde and converts nitroarenes to anilines via transfer hydrogenation. The latter then undergo condensation with formaldehyde providing an *N*-phenylmethanimine intermediate which was confirmed by ^1^H NMR spectroscopy. In the final step, the imine undergoes hydrogenation with **Mn3-b** to yield the N-methylated product (Scheme 16).

**Scheme 17:**
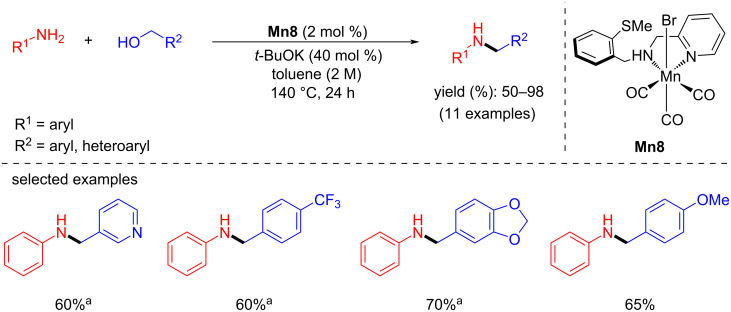
Bidentate manganese complex **Mn8** applied for the N-alkylation of primary anilines with alcohols. ^a^One equiv of *t*-BuOK was used.

In 2020, Maji et al. synthesized manganese(I) complexes bearing bidentate amine-based ligands and studied them in the N-alkylation of aromatic amines with benzylic alcohols (Scheme 17). Under the optimized reaction conditions (140 °C, 24 h), complex **Mn8** (2 mol %) was successfully applied for the coupling of various electron-donating and withdrawing primary amines and aromatic alcohols in the presence of *t*-BuOK (40 mol %) in toluene to give the corresponding secondary amines with up to 98% yield [45].

**Scheme 18:**
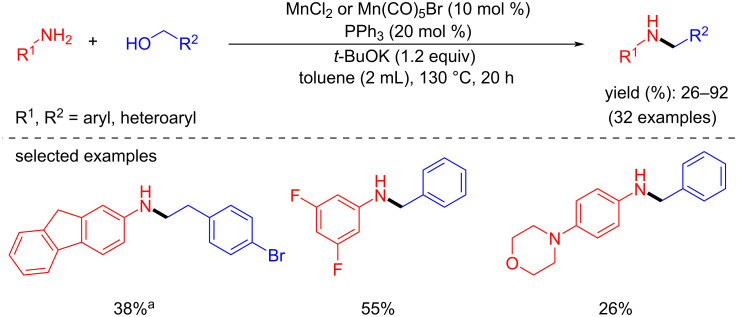
N-Alkylation of amines with alcohols in the presence of manganese salts and triphenylphosphine as the catalytic system. ^a^10 mol % Mn(CO)_5_Br was used.

In 2021, Peng and co-workers demonstrated a practical and operationally simple approach for the N-alkylation of aromatic amines with alcohols using inexpensive and commercially available manganese salts such as MnCl_2_ or Mn(CO)_5_Br and triphenylphosphine (PPh_3_) as ligand [46]. Using this catalytic system (10 mol % Mn precursor, 20 mol % PPh_3_, 1.2 equiv *t-*BuOK, 130 °C, 20 h), a variety of (hetero)aromatic and aliphatic amines were selectively alkylated in moderate-to-high yields with aliphatic and aromatic alcohols (Scheme 18). In addition, this protocol allowed for the synthesis of indole through an intramolecular reaction and a resveratrol-derived amine. However, this catalytic method did not tolerate some functional groups such as nitro, ester, and hydroxy groups and it did not need a prior synthesis of molecularly defined manganese complexes.

**Scheme 19:**
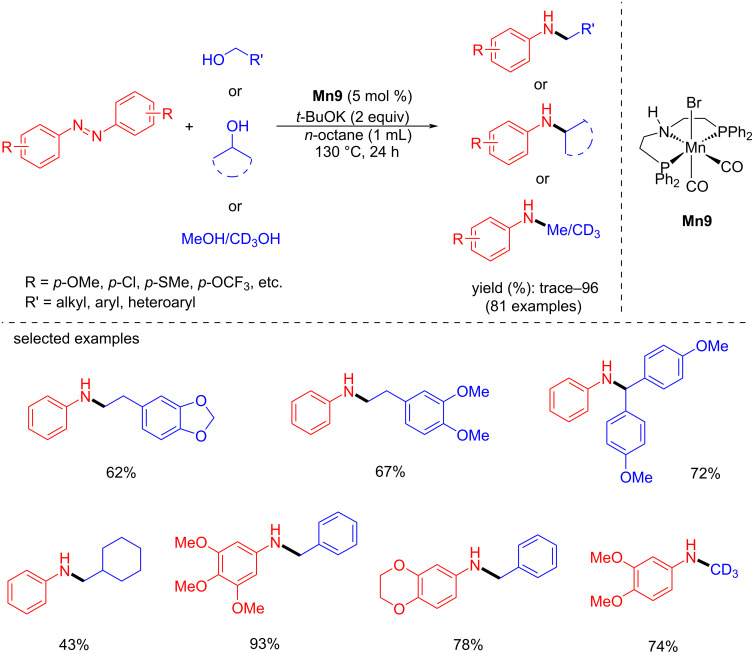
N-Alkylation of diazo compounds with alcohols using catalyst **Mn9**.

Recently, Balaraman’s group introduced a new method for N-alkylation with diazo compounds as an amine source and alcohols as alkylating agents via a tandem process using a manganese(I)-PNP pincer complex [47]. Symmetrical, unsymmetrical, and cyclic azoarenes were studied with benzyl alcohol using catalyst **Mn9** (5 mol %) and *t-*BuOK (2 equiv) at 130 °C for 24 h in octane, resulting in the corresponding N-alkylated amines with up to 96% yield (Scheme 19). On the other hand, various aromatic and aliphatic primary and secondary alcohols were studied with diazobenzene compounds under the same reaction conditions. Remarkably, the N-methylation was carried out with methanol and deuterated methanol and afforded N-methylated/deuterated products with good yields.

**Scheme 20:**
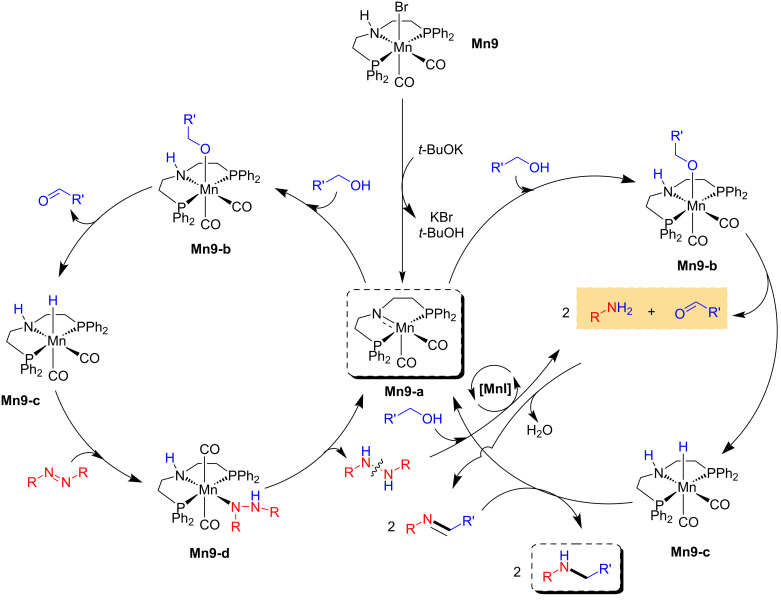
Proposed mechanism for the amination of alcohols with diazo compounds catalyzed by catalyst **Mn9**.

The proposed catalytic cycle showed the formation of the amido complex **Mn9-a** via the debromination of the pre-catalyst **Mn9** with the help of a strong base. Then, **Mn9-a** treated with an alcohol provided a manganese alkoxy complex **Mn9-b,** which then undergoes β-hydride elimination to give the manganese hydride complex **Mn9-c** and the corresponding aldehyde. Further, the azo compound coordinates with the hydride complex **Mn9-c** to give **Mn9-d,** from which the hydrazo compound is released with regeneration of the active amido species **Mn9-a**. Next, the semi-hydrogenated hydrazo compound further undergoes complete hydrogenation. It provides the amine compounds, which condense with aldehydes, leading to the corresponding imine intermediate, which again undergoes hydrogenation by **Mn9-c** and yield the N-alkylated product and the regeneration of complex **Mn9-a** (Scheme 20).

**Scheme 21:**
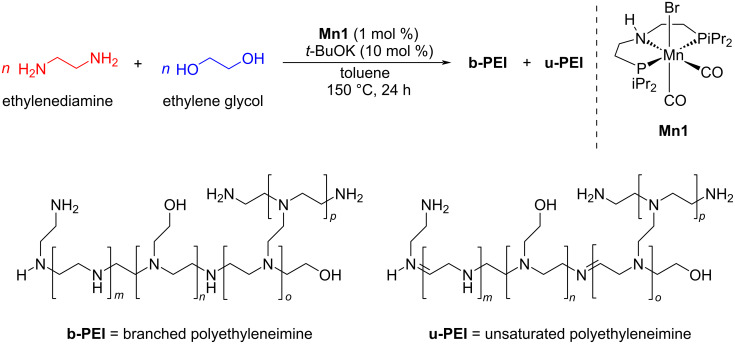
**Mn1** complex-catalyzed synthesis of polyethyleneimine from ethylene glycol and ethylenediamine.

Very recently, Bühl and Kumar reported a novel and efficient methodology for the synthesis of branched polyethyleneimine derivatives by coupling ethylene glycol and ethylenediamine using manganese-pincer catalyst **Mn1** (1 mol %), *t*-BuOK (10 mol %) in toluene at 150 °C (Scheme 21) [48]. The mechanistic investigation based on the experimental and DFT calculations suggested a BH pathway. First, dehydrogenation of the ethylene glycol followed by condensation with ethylenediamine generated the corresponding imine intermediates. The subsequent hydrogenation with borrowed hydrogen finally formed the polyethyleneimine product.

**Scheme 22:**
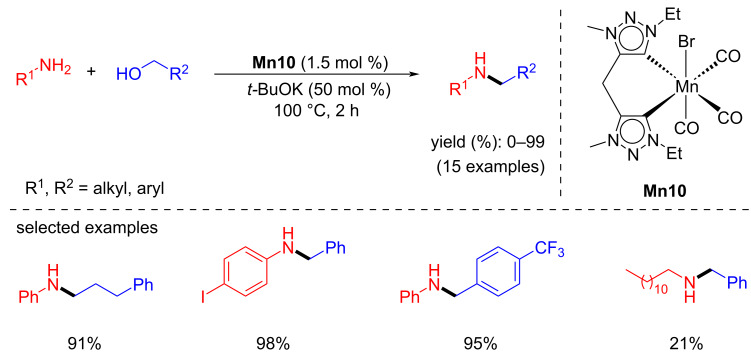
Bis-triazolylidene-manganese complex **Mn10** for the N-alkylation of amines with alcohols.

In 2023, Royo and co-workers conveyed the N-alkylation of amines with alcohols using the bis-triazolylidene manganese complexes [49]. Complex **Mn10** showed superior activity with low catalyst loading (1.5 mol %) and base (50 mol % of *t*-BuOK) at 100 °C for 2 h to afford the N-alkylated products (Scheme 22). Under this protocol, several substituted amines were N-alkylated with various benzyl and aliphatic alcohols and afforded a good to excellent yield. Unfortunately, aliphatic amines such as isopropylamine and cyclohexylamine showed poor activity.

### C–C Bond formation via borrowing hydrogen

Building C–C bonds by selective, efficient, and environmentally benign processes has been challenging and the most commonly used reaction in synthetic chemistry [50–51]. The selective α-functionalization of carbonyl compounds with organohalides in the presence of bases is one of the most fundamental reactions. This methodology usually suffers from the use of stoichiometric amounts of bases and the use of halides, which leads to the formation of a considerable amount of waste [52–54]. The BH approach allows a sustainable way for building C–C bonds by coupling abundant and cheap alcohols with ketones, nitriles, esters, and amides [4].

#### C–C Bond formation via alkylation of ketones with alcohols

**Figure 2:**
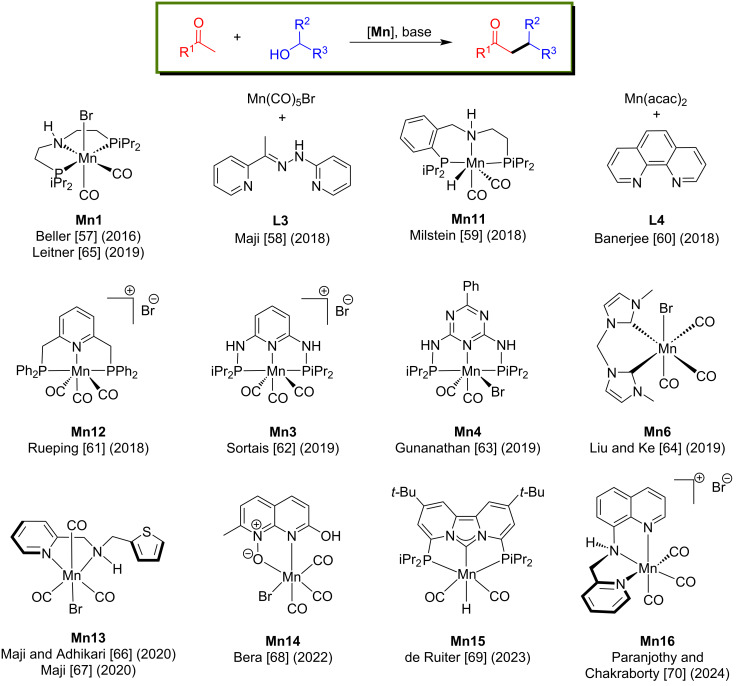
Manganese complexes applied for C-alkylation reactions of ketones with alcohols.

Several homogeneous catalysts, including noble and non-noble metals, have been studied for the alkylation of ketones with primary and secondary alcohols [55–56]. In this section, we discuss the development of manganese complexes (Figure 2) for coupling primary and secondary alcohols with ketones to give the corresponding alkylated ketones or alcohols.

**Scheme 23:**
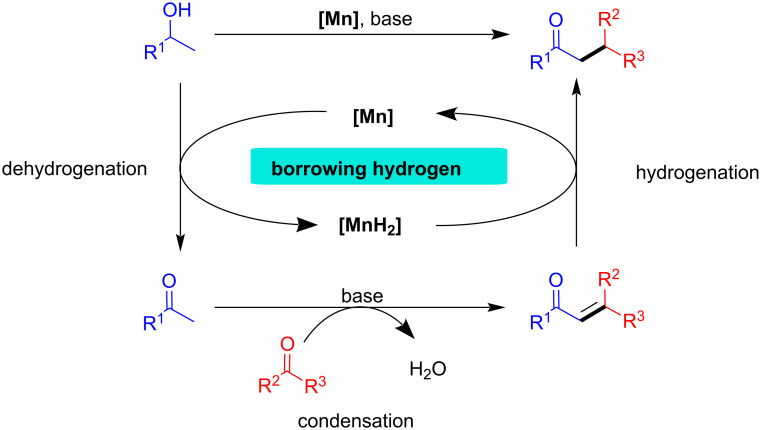
General scheme for the C–C bond formation with alcohols and ketones.

As depicted in the general Scheme 23, in the first step, the manganese-catalyzed dehydrogenation of alcohols delivers carbonyl compounds, which condense with other carbonyl compounds in the presence of a base to afford unsaturated intermediate compounds. In the final step, reduction of unsaturated compounds with manganese hydride complexes giving the desired C-alkylated products.

**Scheme 24:**
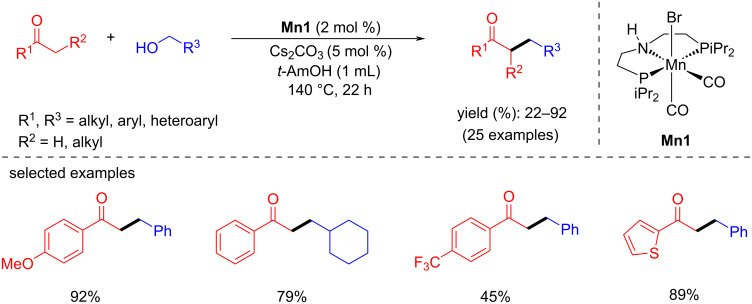
**Mn1** complex-catalyzed α-alkylation of ketones with primary alcohols.

In 2016, Beller and co-workers introduced the first air-stable manganese(I)-PNP-pincer pre-catalyst for the α-alkylation of ketones with primary alcohols [57]. The reaction conditions were investigated using four different well-defined phosphine substituents-containing Mn complexes with acetophenone and benzyl alcohol as model substrates. Among these, complex **Mn1** showed better results with 2 mol % loading and at low base concentration (Cs_2_CO_3_; 5 mol %) in *tert*-amyl alcohol at 140 °C for 22 h, giving 88% yield of the desired alkylated product. Several ketones were studied under the same conditions, with substituted benzyl and aliphatic alcohols giving up to 92% yield of the corresponding C-alkylated products (Scheme 24).

**Scheme 25:**
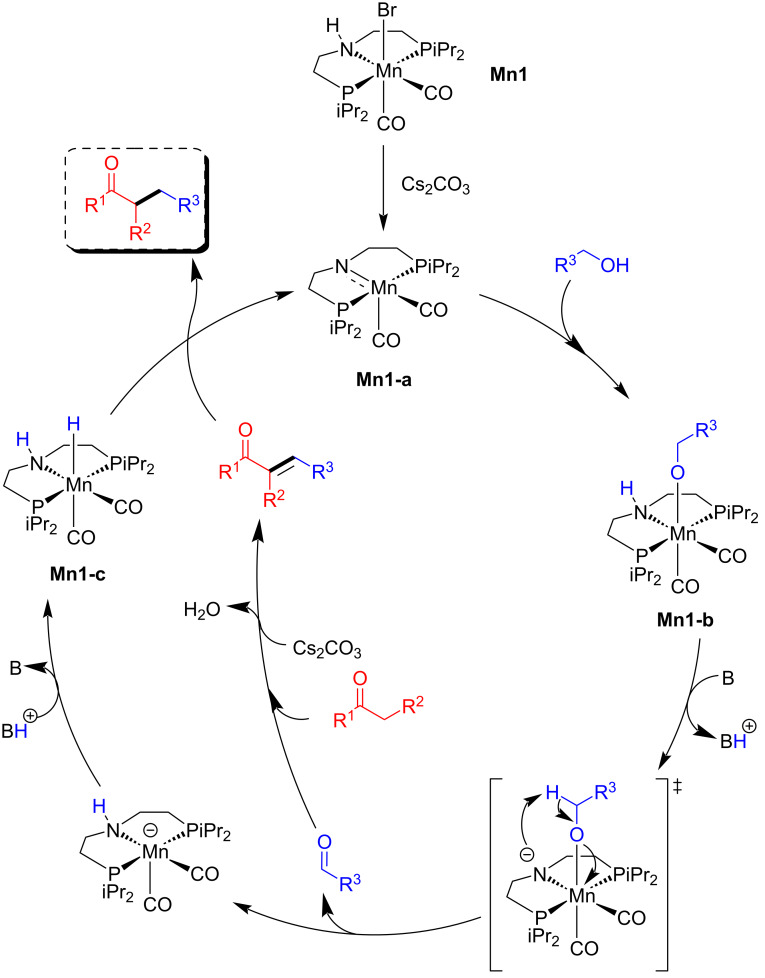
Mechanism for the **Mn1**-catalyzed alkylation of ketones with alcohols.

The proposed mechanism showed that the pre-catalyst **Mn1** was first activated by the base, affording the active amido complex **Mn1-a** which reacts with the alcohol to form the alkoxo-type complex **Mn1-b**. An intramolecular ligand-assisted mechanism produced the aldehyde and manganese hydride complex **Mn1-c** after protonation of the intermediate. The aldehyde then underwent aldol condensation with ketones, yielding an α,β-unsaturated compound, which was hydrogenated by the manganese hydride species, resulting in the final alkylated product (Scheme 25). A set of deuterium labelling tests and additional control studies determined that the alcohol dehydrogenation was aided by an intramolecular manganese amidate rather than the traditional β-hydride elimination process.

**Scheme 26:**
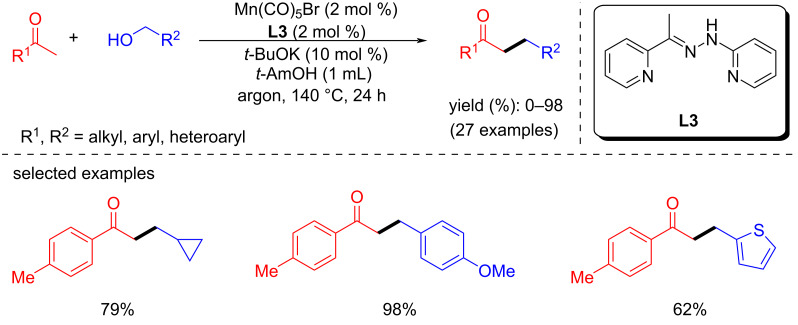
Phosphine-free in situ-generated manganese catalyst for the α-alkylation of ketones with primary alcohols.

In 2018, Maji’s group reported the α-alkylation of ketones with primary alcohols using a phosphine-free manganese catalyst generated in situ from Mn(CO)_5_Br and **L3** [58]. Under optimized conditions (2 mol % Mn(CO)_5_Br, 10 mol % *t*-BuOK, *t*-AmOH, argon atmosphere), various substituted ketones were selectively alkylated with benzyl alcohols as alkyl source and hydrogen donor at 140 °C for 24 h and afforded up to 98% yield of the C-alkylated products (Scheme 26). In addition, numerous substituted benzylic, aliphatic, and heterocyclic alcohols were tested and showed good functional group tolerances. However, ester and nitrile-substituted ketones were not alkylated with this protocol.

**Scheme 27:**
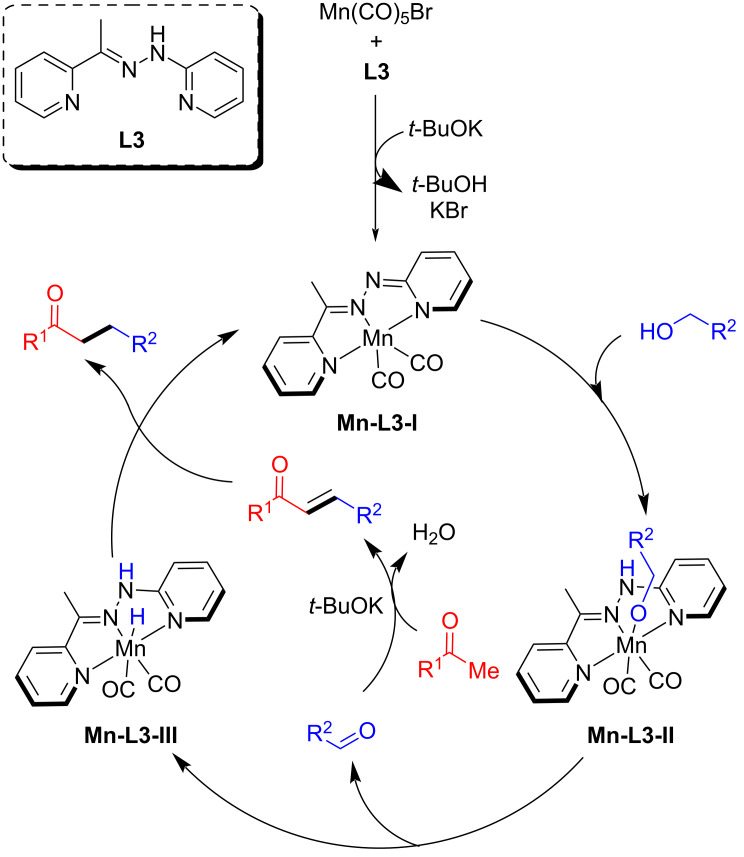
Plausible mechanism for the Mn-catalyzed α-alkylation of ketones with alcohols.

The proposed mechanism showed that the Mn(CO)_5_Br reacted with ligand **L3** to generate the active complex **Mn-L3-I** in the presence of a base. The formed active catalyst dehydrogenates the alcohol to generate the alkoxy complex **Mn-L3-II**. The liberated aldehyde undergoes aldol condensation with the ketone to afford the α,β-unsaturated ketone, followed by the selective hydrogenation with **Mn-L3-III** to give the desired alkylated product (Scheme 27).

**Scheme 28:**
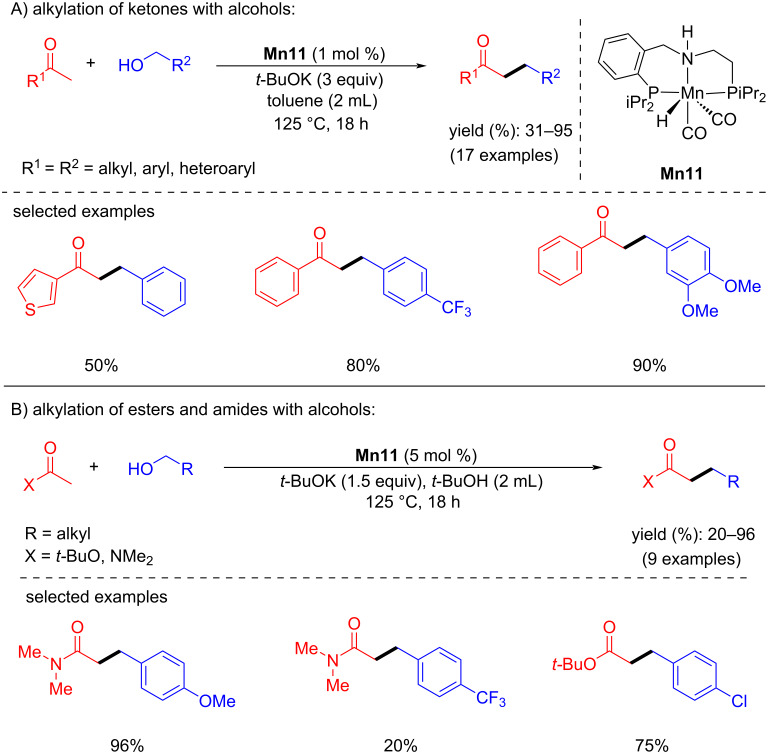
α-Alkylation of esters, ketones, and amides using alcohols catalyzed by **Mn11**.

In the same year, Milstein and co-workers accomplished the α-alkylation of esters, ketones, and amides using alcohols as alkylating agents with a PNP-pincer-supported manganese catalyst [59]. First, the α-alkylation of ketones with benzylic and aliphatic alcohols was studied using **Mn11** (1 mol %) as catalyst and *t*-BuOK as a base at 125 °C for 18 h in toluene which afforded up to 95% yield of the desired alkylated ketones (Scheme 28A). Later, the more challenging esters and amides were selectively alkylated with alcohols, however, required higher catalyst loading (5 mol %) and a stoichiometric amount of *t*-BuOK (1.5 equiv) at 125 °C for 18 h (Scheme 28B). The proposed mechanism suggested the formation of α,β-unsaturated ketones as the intermediates, similar to the previous report [58] and the selective hydrogenation of the C=C bond was the last step.

**Scheme 29:**
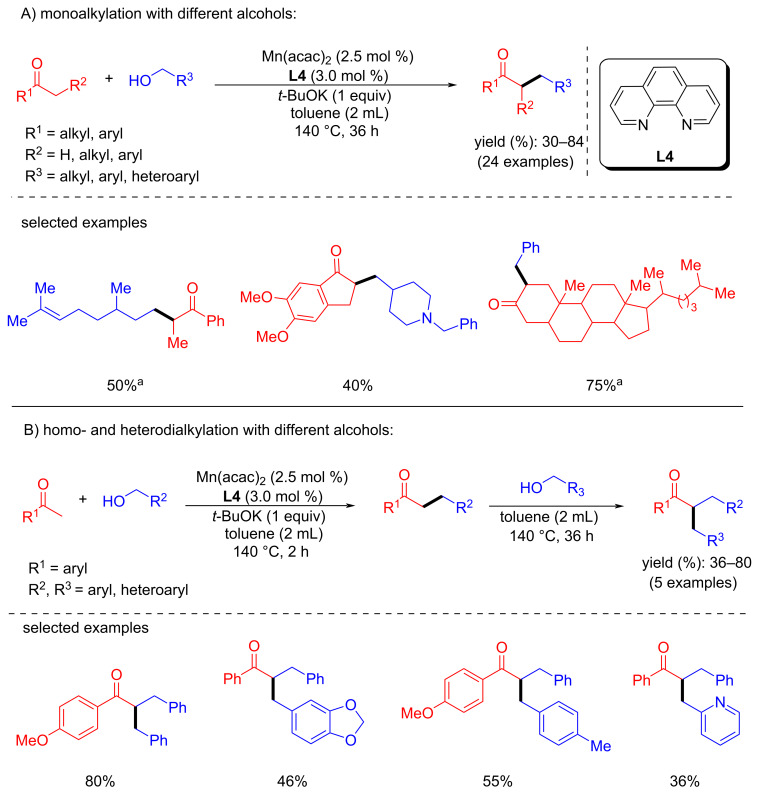
Mono- and dialkylation of methylene ketones with primary alcohols using the Mn(acac)_2_/1,10-phenanthroline system. ^a^24 h reaction time.

In 2018, Banerjee’s group developed the alkylation of methylene ketones with primary alcohols using a phosphine-free and commercially available Mn(acac)_2_/1,10-phenanthroline system [60]. Various methylene ketones and alcohols were investigated with Mn(acac)_2_ (2.5 mol %) as a precursor, 1,10-phenanthroline (3 mol %) as ligand, and *t*-BuOK (1 equiv) as a base in toluene at 140 °C for 36 h that gave up to 84% yield (Scheme 29A). More interestingly, double alkylation also occurred in one pot using acetophenone and 4-methoxyacetophenone with different benzyl alcohols under the optimized conditions. In the first step, monoalkylation of the methyl ketone led to the linear α-alkylated product, followed by the alkylation of the methylene ketone with the second benzyl alcohol then afforded the dialkylated product. Remarkably, the drug donepezil, a steroid derivative and a fatty acid derivative were synthesized using this procedure (Scheme 29B).

**Scheme 30:**
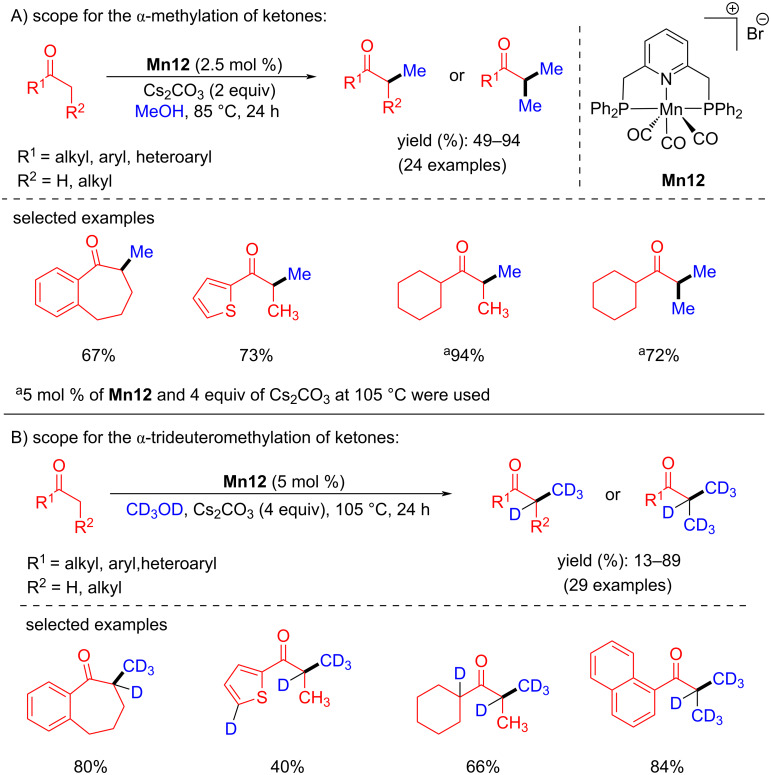
Methylation of ketones with methanol and deuterated methanol.

In 2018, Rueping and co-workers reported a manganese-catalyzed α-methylation of ketones with methanol and deuterated methanol. Many ketones were investigated with methanol under the optimized conditions (2.5 mol % of **Mn12**, 2 equiv of Cs_2_CO_3_, 85 °C for 24 h) providing yields up to 94% [61]. Interestingly, trideuteromethylation of ketones were studied with 5 mol % of **Mn12** and 4 equiv of Cs_2_CO_3_ at 105 °C for 24 h giving up to 89% yield. More interestingly, the double trideuteromethylation of ketones was also reported (Scheme 30).

**Scheme 31:**
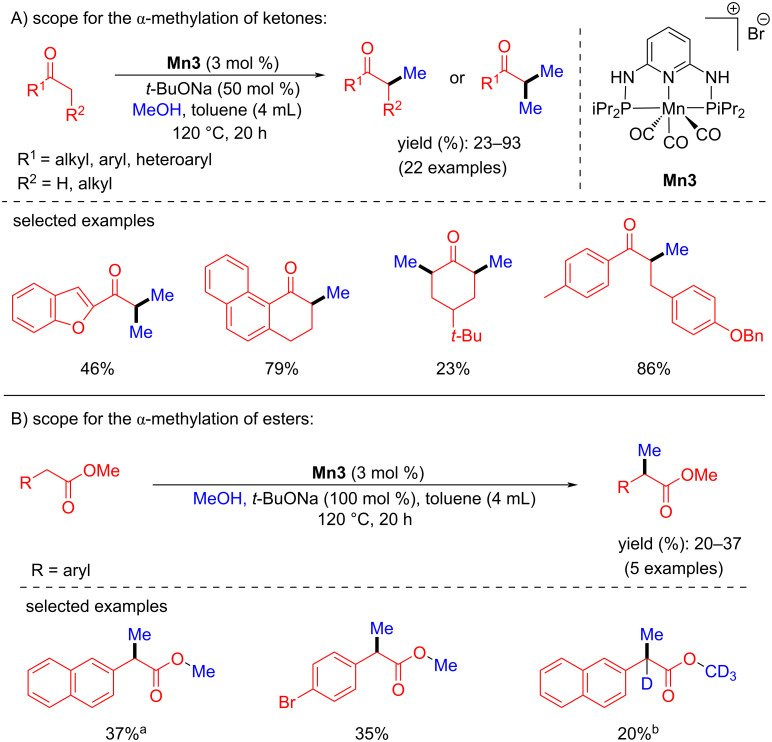
Methylation of ketones and esters with methanol. ^a^50 mol % of *t*-BuOK were used, ^b^CD_3_OD was used instead of CH_3_OH, 48 h.

In 2019, Sortais reported the α-methylation of several ketones with methanol as a C1 source. Under the optimized reaction conditions, they achieved good yields (23–93%) of the desired methylated products that could be obtained with 3 mol % catalyst loading and 50 mol % *t*-BuONa in a closed pressure tube at 120 °C for 20 h [62]. In addition, the α-methylation of esters was studied under the optimized conditions with 100 mol % of the base, however, only a poor yield was obtained (Scheme 31).

**Scheme 32:**
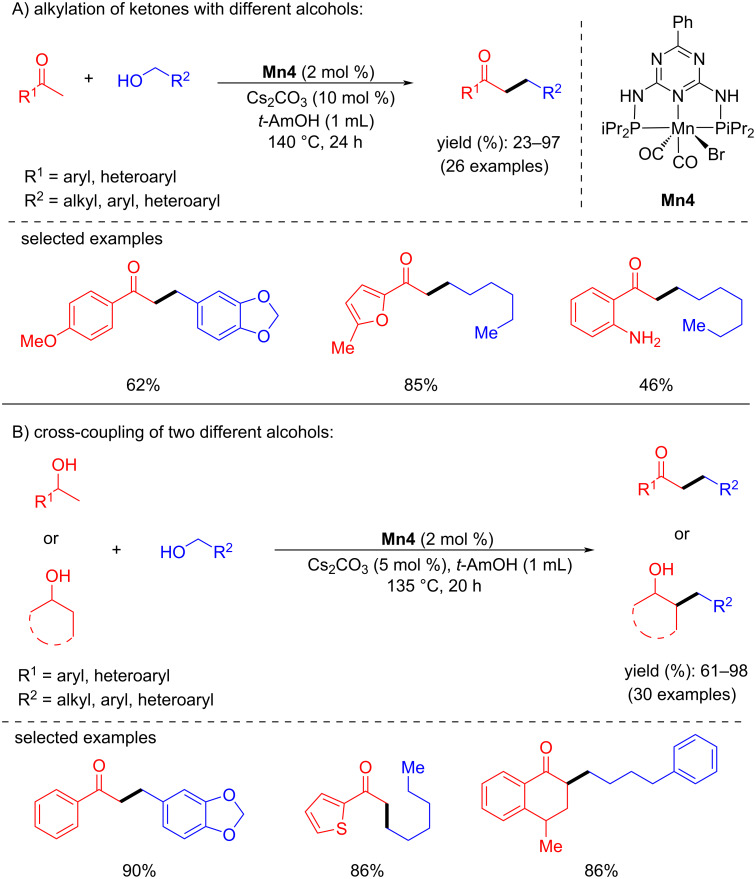
Alkylation of ketones and secondary alcohols with primary alcohols using **Mn4**.

Later, Gunanathan and co-workers reported an efficient method for the chemoselective alkylation of ketones and secondary alcohols with primary alcohols using a Mn(I)-PNP pincer complex [63]. The reaction of different ketones with several alcohols (aliphatic and benzylic) was carried out at 140 °C for 24 h in the presence of 2 mol % **Mn4** and 10 mol % of Cs_2_CO_3_ and provided up to 97% of the desired alkylated products. Notably, the alkylation of ketones using ethanol as a coupling partner was also established. Furthermore, β-alkylation of 1-phenyl-1-ethanol with benzylic alcohols was also studied with 2 mol % of the **Mn4** pre-catalyst, 5 mol % of Cs_2_CO_3_ in *t*-AmOH at 135 °C for 20 h (Scheme 32). NMR studies endorsed the formation of intermediates such as aldehyde, ketone, and α,β-unsaturated ketone. The proposed mechanism suggested that dearomatization–aromatization pathways operated for the dehydrogenation of the alcohol and C–C bond formations.

**Scheme 33:**
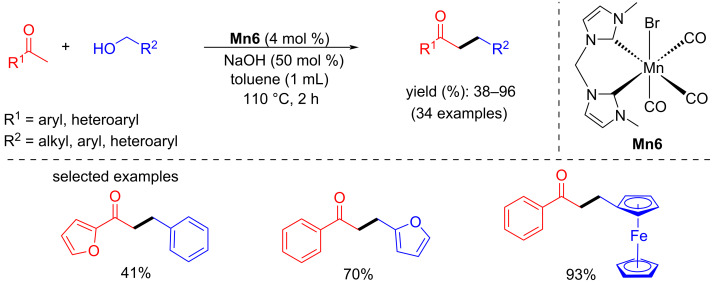
Bidentate manganese-NHC complex **Mn6** applied for the synthesis of alkylated ketones using alcohols.

After the successful attempt of bidentate N-heterocyclic carbene-manganese complex-catalyzed N-alkylation of amines with alcohols at room temperature [41], Liu and Ke's group planned the α-alkylation of ketones using alcohols as an alkylating agent [64]. A number of substituted aromatic and heterocyclic ketones with different alcohols were tested and gave good to excellent yields (38–96%) using 4 mol % of **Mn6** and 50 mol % of NaOH in toluene at 110 °C for 2 h (Scheme 33). The reaction proceeded via the dehydrogenation of the alcohol, aldol condensation, and hydrogenation of α,β-unsaturated ketones.

**Scheme 34:**
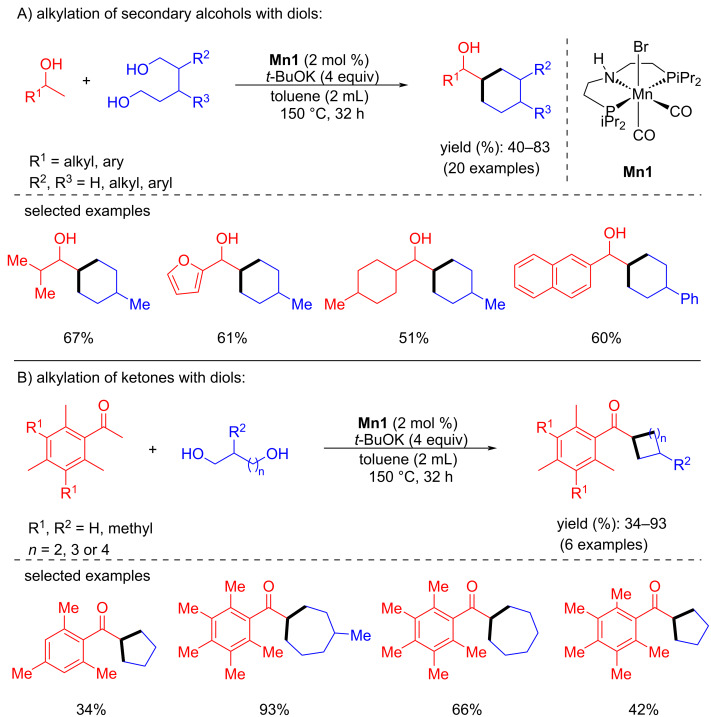
**Mn1**-catalyzed synthesis of substituted cycloalkanes by coupling diols and secondary alcohols or ketones.

In 2019, Leitner and his group introduced an outstanding cascade BH approach for the synthesis of various substituted cycloalkanes by coupling diols and secondary alcohols or ketones [65]. Various substituted secondary alcohols were treated with 1,5-pentanediol in the presence of 2 mol % of **Mn1** and 4 equiv of *t*-BuOK as base in toluene at 150 °C for 32 h to afford the desired products with moderate to good yields (40–83%). In addition, five to seven-membered rings were constructed by treating aromatic ketones with substituted diols. However, a stoichiometric amount of the base (4 equiv), excess of diols (4 equiv), and long reaction time (32 h) were required to deliver the desired products (Scheme 34).

**Scheme 35:**
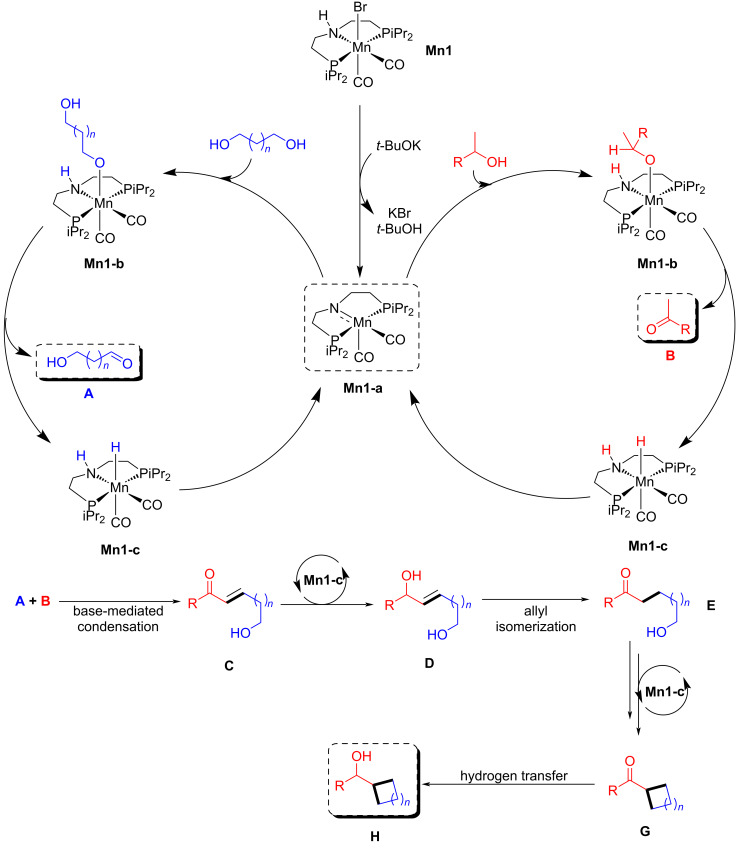
Proposed mechanism for the synthesis of cycloalkanes via BH method.

The proposed mechanism demonstrated that the active amido complex **Mn1-a** dehydrogenated secondary alcohols into ketone **B** and diol into aldehyde **A**. Further, aldol condensation occurred between the ketone and aldehyde and produced α,β-unsaturated ketone **C**, which was subsequently hydrogenated by complex **Mn1-c**, followed by allyl isomerization, which led to the formation of hydroxy ketone compound **E**. Cyclization occurred via dehydrogenation and intramolecular aldol condensation and in the last step hydrogen transfer provided the desired cyclic product **H** (Scheme 35).

**Scheme 36:**
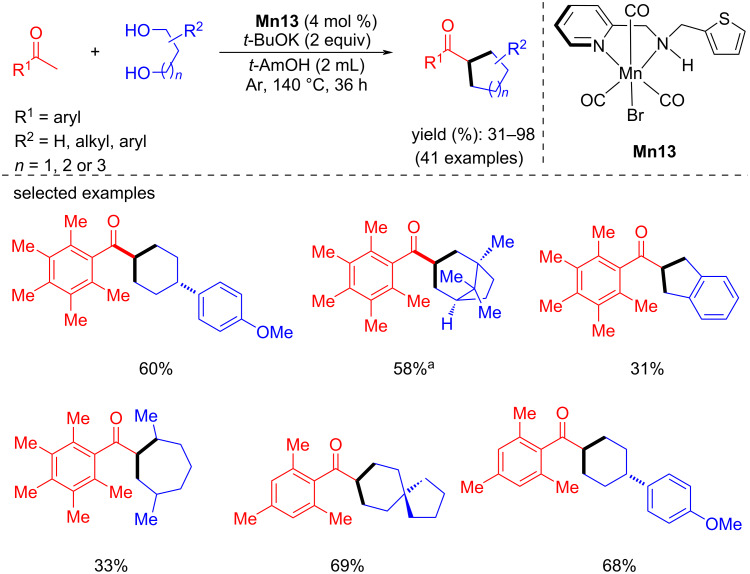
Synthesis of various cycloalkanes from methyl ketones and diols catalyze by **Mn13**. ^a^Reaction time was 6 h.

In 2020, Maji and Adhikari reported a phosphine-free *N*,*N*-amine–manganese complex-catalyzed stereoselective intermolecular and intramolecular BH reaction for the formation of cycloalkanes from ketones and 1,*n*-diols [66]. Different substituted 1,5-diols were coupled with 1-(2,3,4,5,6-pentamethylphenyl)ethan-1-one using 4 mol % of catalyst **Mn13** and 2 equiv of *t*-BuOK as base in *t*-AmOH at 140 °C for 36 h to give 51–98% yield of the six-membered ring products. They isolated cyclic five to seven-membered ring products by changing the lengths of the diols. For example, for the formation of cyclopentane products, butane-1,4-diol was used as the alcohol under the same reaction conditions, giving 31 to 70% yield of the desired products. Seven-membered rings were also formed only by changing the alcohols to hexane-1,6-diol under the same conditions as above, giving yields up to 80%. In addition, several ketones were investigated under these conditions with different diol systems, giving 55–80% yields of the cyclic products (Scheme 36). DFT studies showed that the hemilability and bifunctionality of the thiophene arm attached to the metal play an important role in this transformation for the dehydrogenation and hydrogenation steps.

**Scheme 37:**
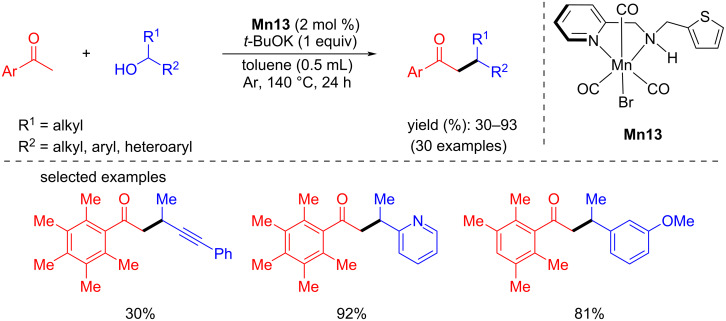
*N*,*N*-Amine–manganese complex (**Mn13**)-catalyzed alkylation of ketones with alcohols.

Later, the same complex was successfully used for the α-alkylation of ketones with secondary alcohols to synthesize β-branched carbonyl compounds [67]. The reaction conditions were optimized by treating the 2,3,4,5,6-pentamethylacetophenone with cyclohexanol by different manganese complexes. Among all the complexes, 2 mol % of **Mn13** and one equiv of *t*-BuOK as base in toluene at 140 °C for 24 h under an argon atmosphere afforded 85% yield of the desired alkylated product. Pentamethylacetophenone was alkylated with several secondary alcohols, giving yields between 30 and 93%, which included aliphatic and heteroaryl secondary alcohols (Scheme 37).

**Scheme 38:**
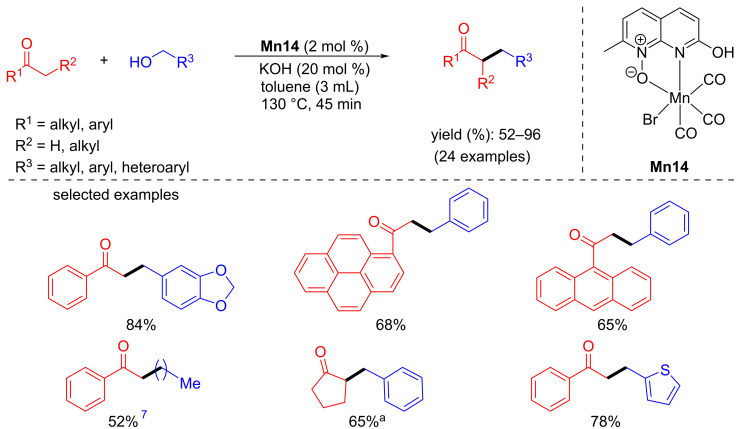
Naphthyridine‑*N*‑oxide manganese complex **Mn14** applied for the alkylation of ketones with alcohols. ^a^Reaction time was 6 h.

**Scheme 39:**
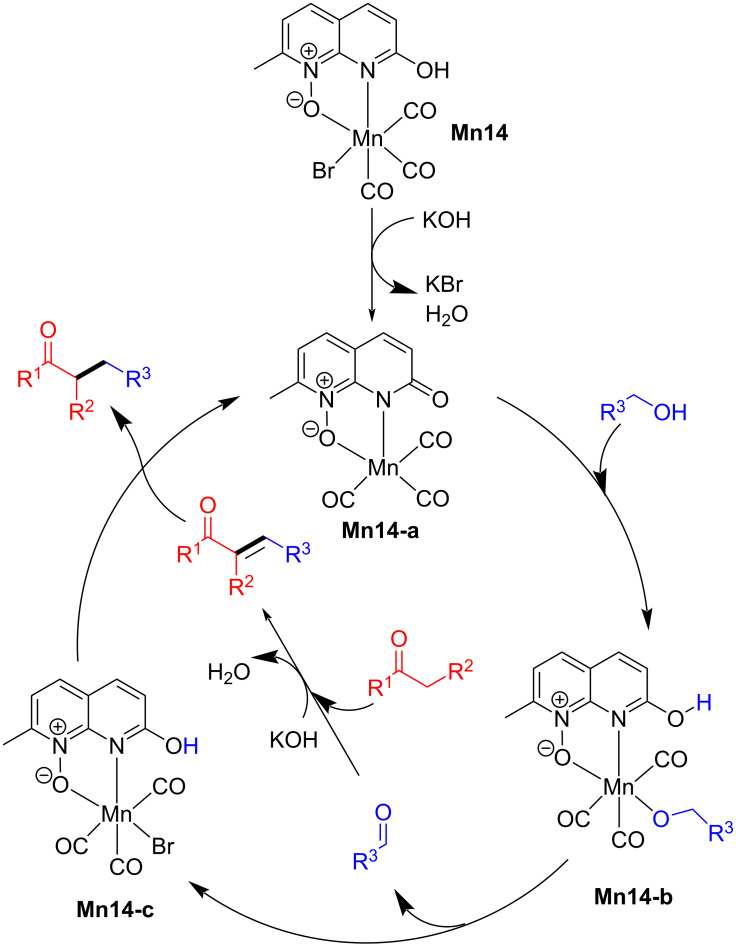
Proposed mechanism of the naphthyridine‑*N*‑oxide manganese complex (**Mn14**)-catalyzed alkylation of ketones with alcohols.

**Scheme 40:**
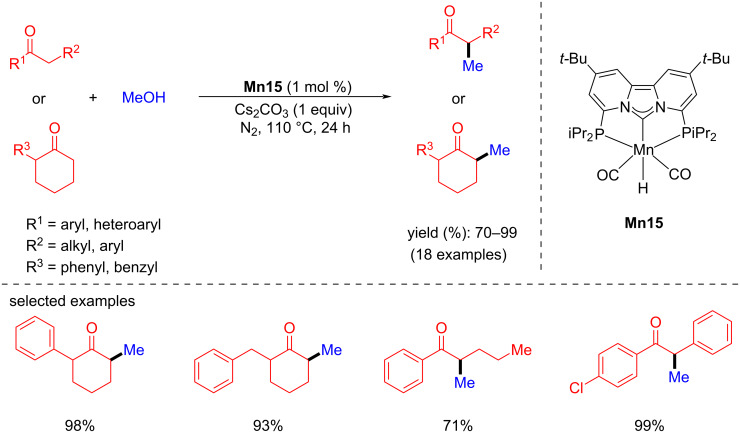
α-Methylation of ketones and indoles with methanol using **Mn15**.

In 2022, Bera’s group reported the α-alkylation of ketones with alcohols as an alkylating agent using the protic functionality on a naphthyridine‑*N*‑oxide manganese complex [68]. The reaction conditions were optimized using acetophenone and benzyl alcohol as model substrates. After optimizing various reaction parameters, 2 mol % of **Mn14** and 20 mol % of KOH in toluene afforded the α-alkylated product with 96% yield. Under the optimized catalytic conditions, various substituted alcohols were investigated with acetophenone, which gave good to excellent yields of the alkylated products (Scheme 38). The scope of ketones was also tested with benzyl alcohols, which gave yields up to 85%. The proposed mechanism suggested the formation of the dehydrogenation product and the desired product due to the metal–ligand cooperation (Scheme 39).

de Ruiter and co-workers studied PCNHCP-based manganese complexes for the α-methylation of ketones and indoles with methanol as a C1 source in 2023 [69]. The reaction conditions were optimized using three different Mn catalysts and bases. Among them, complex **Mn15** gave the better yield with 1 mol % of **Mn15**, Cs_2_CO_3_ (1 equiv) as a base in methanol at 110 °C for 24 h under N_2_ atmosphere, giving 99% of the methylated product. The same conditions were followed for the methylation of several ketones with methanol, which gave yields of up to 99% (Scheme 40).

**Scheme 41:**
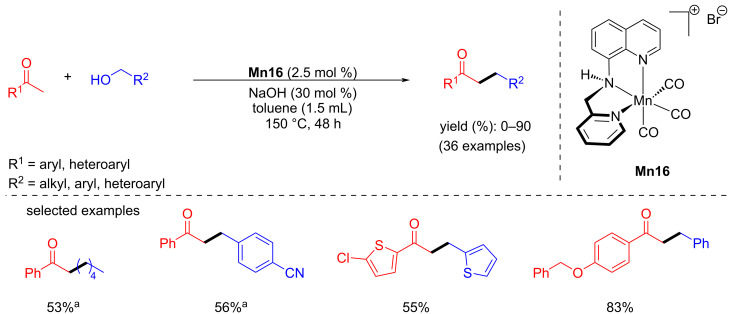
α-Alkylation of ketones with primary alcohols using **Mn16**. ^a^NMR yield.

Very recently, a quinoline-based manganese catalyst was studied by Chakraborty and co-workers for the alkylation of methyl aryl ketones with alcohols (Scheme 41) [70]. Several methyl ketones and alcohols were studied using 2.5 mol % of **Mn16** and 30 mol % of NaOH in toluene and yields up to 90% were achieved at high temperature (150 °C) and long reaction time (48 h).

#### C–C Bond formation through coupling of various alcohols

**Figure 3:**
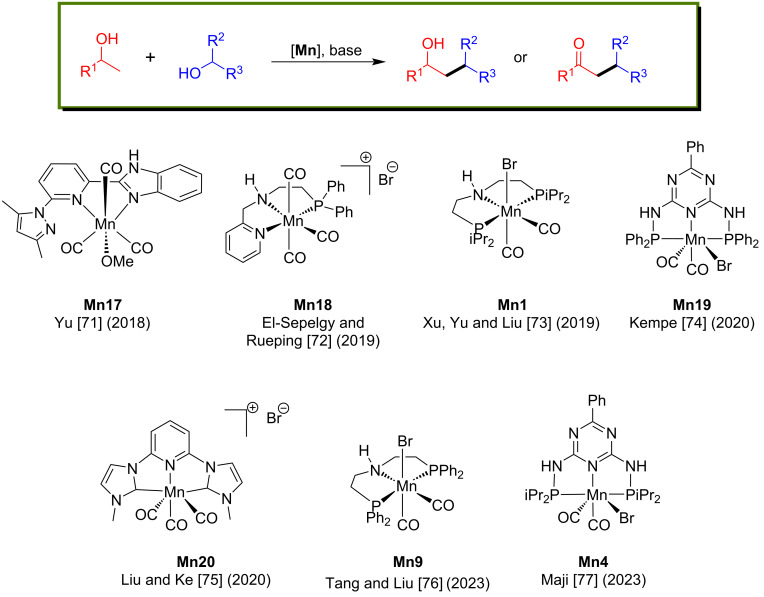
Manganese complexes used for coupling of secondary and primary alcohols.

Cross-coupling of two different alcohols to build C–C bonds is challenging because of the formation of undesired side products from aldol condensation. Herein, we summarized the reported manganese complexes applied for the coupling of secondary and primary alcohols to form a C–C bond (Figure 3).

**Scheme 42:**
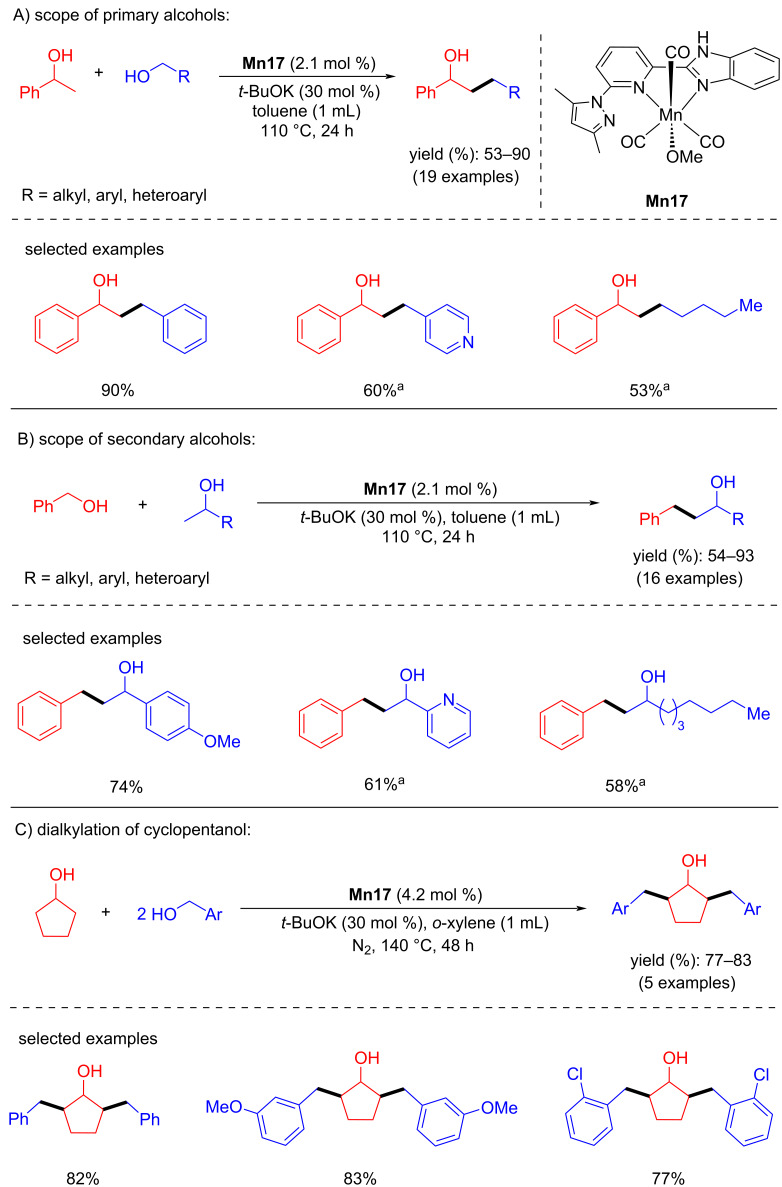
Alkylation of secondary alcohols with primary alcohols catalyzed by phosphine-free catalyst **Mn17**. ^a^4.2 mol % of **Mn17** were used.

In 2018, Yu’s group introduced phosphine-free manganese(I) catalytic systems for the direct β-alkylation of secondary alcohols with primary alcohols [71]. The reaction conditions were investigated for the alkylation of 1-phenylethanol with benzyl alcohol using manganese complexes containing pyridyl-supported pyrazolyl-imidazolyl ligands and bases. Among these complexes, **Mn17** gave an isolated yield of 90% with 2.1 mol % of **Mn17** and 30 mol % of *t*-BuOK in toluene at 110 °C for 24 h. Various benzylic, heteroaromatic, and aliphatic alcohols were reacted with 1-phenylethanol, giving the products with up to 90% yield. Similarly, variation of the secondary alcohols in the reaction with benzyl alcohol gave good to excellent product yields of 54–93%. Interestingly, dialkylated products were achieved when cyclopentanol was treated with benzylic alcohols at 140 °C for 48 h. In addition, 5α-cholestan-3β-ol was also selectively monoalkylated with benzylic alcohols (Scheme 42).

**Scheme 43:**
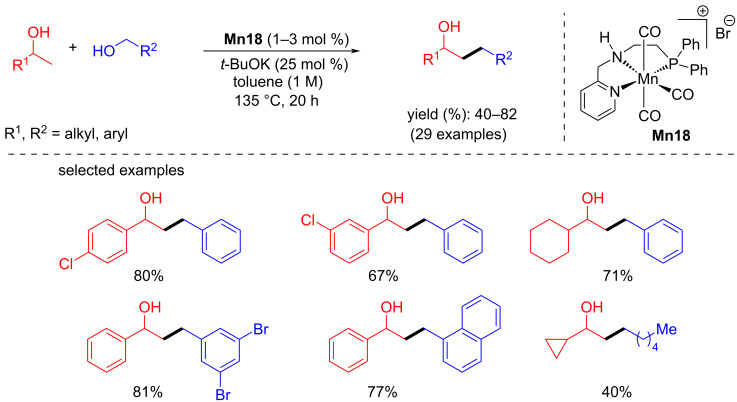
PNN-Manganese complex **Mn18** for the alkylation of secondary alcohols with primary alcohols.

**Scheme 44:**
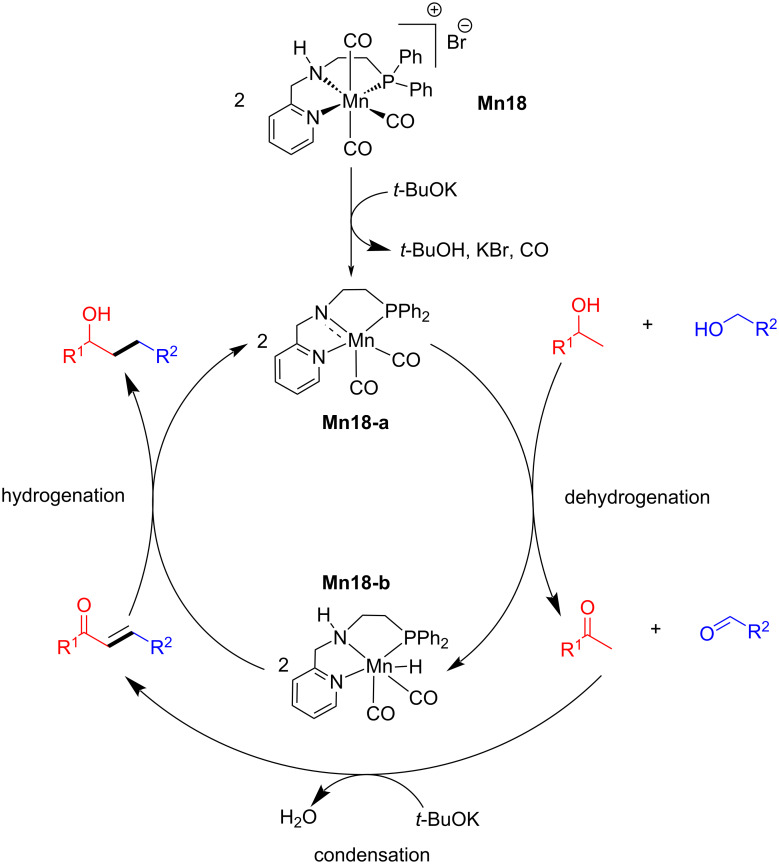
Mechanism for the Mn-pincer catalyzed C-alkylation of secondary alcohols with primary alcohols.

In 2019, El-Sepelgy and Rueping’s team reported that a stable PNN-manganese-pincer complex catalyzed the C-alkylation of secondary alcohols with primary alcohols [72]. Four different manganese catalysts were investigated for the alkylation of 1-phenylethanol with benzyl alcohol. Among these, **Mn18** showed excellent activity with low catalyst loading (1 mol %), *t*-BuOK (25 mol %) as base, and toluene as solvent at 135 °C for 20 h under argon conditions, giving a yield of 82% (Scheme 43). Substituted aromatic and aliphatic secondary alcohols with benzyl alcohol gave 40 to 82% yields, and 1-phenylethanol with substituted primary alcohols gave moderate to good yields (50–82%). Deuterium-labelling experiments with deuterated 1-phenylethanol**-**α*-d*_1_ and benzyl alcohol-α,α-*d*_2_ suggested a hydrogen auto-transfer and dehydrogenation process. The amido species **Mn18-a** generated from **Mn18** by the base is responsible for the dehydrogenation of alcohol-yielding **Mn18-b** species. Mn–H complex reduced the C=C and C=O bonds, yielding the fully reduced saturated alcohol products (Scheme 44).

**Scheme 45:**
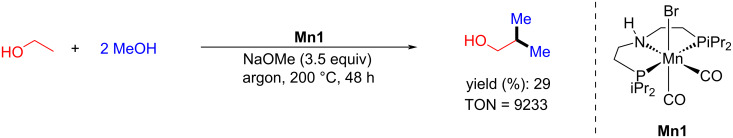
Upgrading of ethanol with methanol for isobutanol production.

In 2019, the upgrading of bio-derived ethanol with widely available methanol for the production of isobutanol was developed by Liu and co-workers using Mn-pincer (PNP) complex **Mn1** at various concentrations. An extraordinary TON (9233) could be achieved at low catalyst loading using 3.5 equiv NaOMe at 200 °C for 48 h with 91% selectivity and 29% yield (Scheme 45) [73].

**Scheme 46:**
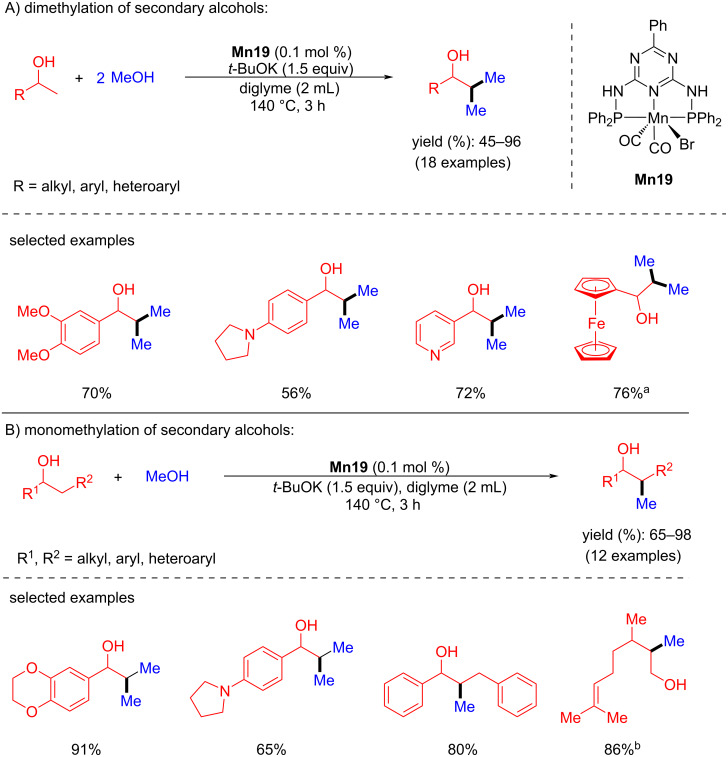
Mn-Pincer catalyst **Mn19** applied for the β-methylation of alcohols with methanol. ^a^2.0 mol % of **Mn19** were used, 12 h. ^b^Reaction time was 6 h.

In 2020, Kempe's group reported an elegant example for the β-methylation of alcohols by methanol as a methylating agent using a manganese PN^5^P pincer complex [74]. First, the double methylation of the primary carbon atoms of various secondary alcohols was investigated with methanol using very low catalyst loading (0.1 mol % of **Mn19**) and 1.5 equiv of *t*-BuOK in diglyme as solvent at 140 °C for 3 h and gave up to 96% yield of the dimethylated products. Interestingly, under the same reaction conditions, monomethylation of the secondary carbon atom of the alcohols afforded up to 98% yield (Scheme 46).

**Scheme 47:**
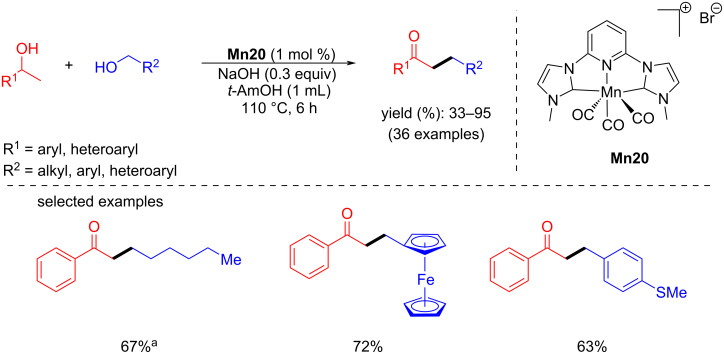
Functionalized ketones from primary and secondary alcohols catalyzed by **Mn20**. ^a^**Mn20** (5 mol %), NaOH (0.5 equiv), 130 °C.

In 2020, Liu and Ke reported that a phosphine-free manganese complex catalyzed the coupling of secondary and primary alcohols for the formation of ketones [75]. The reaction conditions were optimized with 1-phenylethan-1-ol and benzyl alcohols using several manganese complexes. Among all, 1 mol % of **Mn20**, 0.3 equiv of NaOH in *t*-AmOH at 110 °C for 6 h afforded the alkylated ketone with 88% yield. Under the same conditions, several primary alcohols were tested and gave good to excellent yields (up to 95%) of the desired products. Various substituted secondary alcohols were also tested, giving the alkylated ketones in yields up to 87% (Scheme 47).

**Scheme 48:**
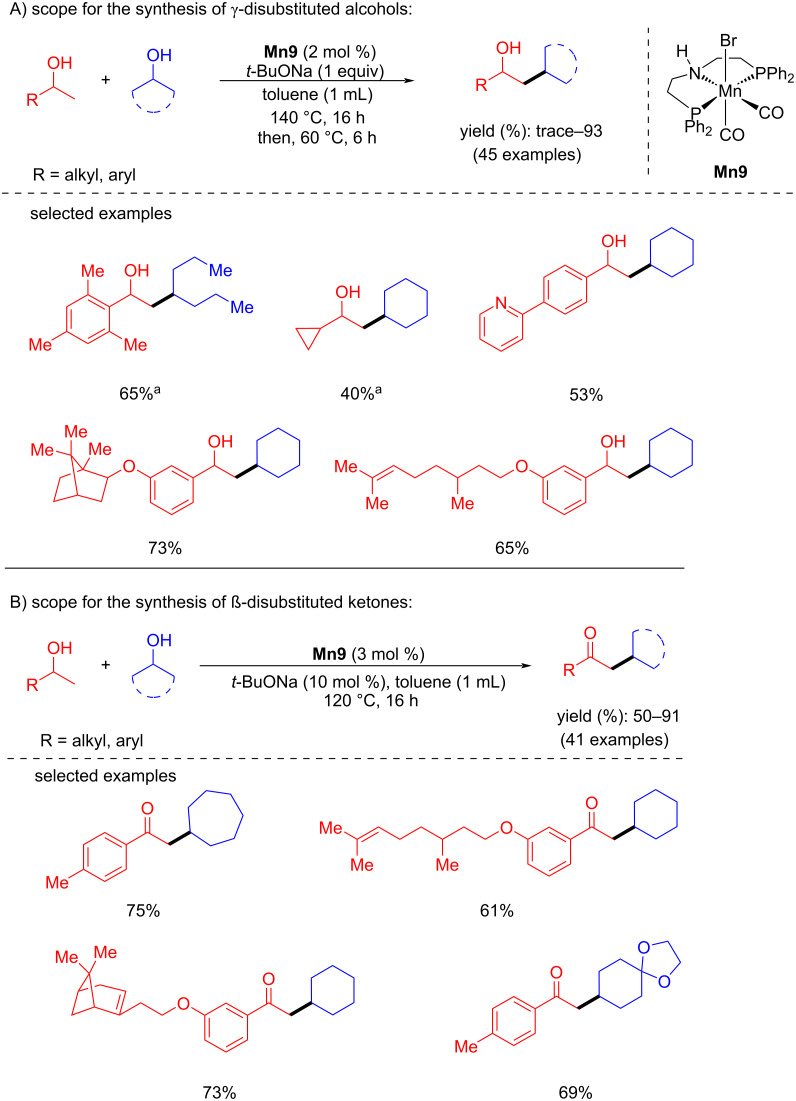
Synthesis of γ-disubstituted alcohols and β-disubstituted ketones through **Mn9**-catalyzed coupling of two secondary alcohols. ^a^4.0 mol % of **Mn9** were used.

Tang and Liu reported the divergent synthesis of γ-disubstituted alcohols and β-disubstituted ketones via coupling the two secondary alcohols [76]. The selectivity was obtained by controlling the reaction conditions. Using the catalyst **Mn9** (2 mol %) and a stoichiometric amount of *t*-BuONa (1 equiv) at 140 °C for 16 h in a sealed system, several γ-disubstituted alcohols were isolated from moderate to high yield (Scheme 48). Notably, the reduced temperature to 60 °C for 6 h is necessary to achieve the excellent yield of the desired γ-disubstituted alcohol products. To access the β-disubstituted ketones from secondary alcohols, 3 mol % of the manganese catalyst, a catalytic amount of *t*-BuONa (10 mol %) and an open reflux system (120 °C in toluene) under argon flow were required. Utilizing this unique method, many aromatic, aliphatic, and acyclic alcohols were cross-coupled, furnishing a library of disubstituted alcohols and ketones in moderate to good yields with good functional group tolerance.

**Scheme 49:**
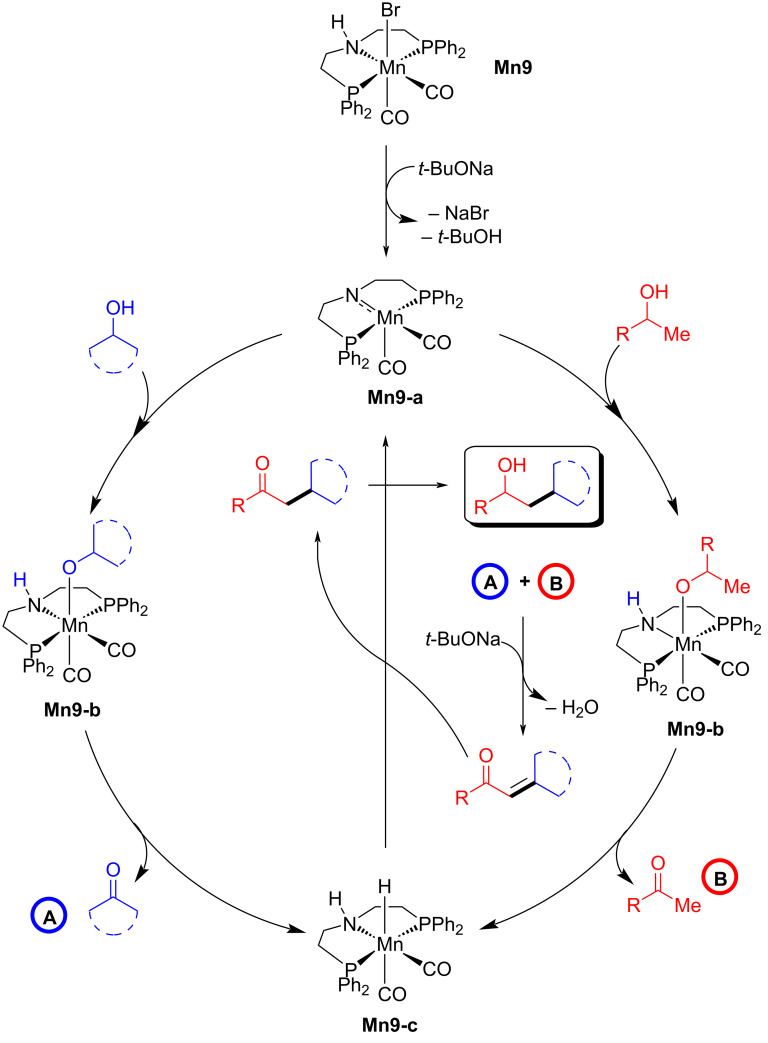
Proposed mechanism for the **Mn9**-catalyzed synthesis of γ-disubstituted alcohols and β-disubstituted ketones.

The proposed mechanism showed that the amido complex dehydrogenated the secondary alcohols into the corresponding carbonyl compounds, which undergo base-assisted aldol condensation, providing the unsaturated ketone compounds. Then, **Mn9-c** hydrogenated the C=C and C=O bonds delivering the desired alkylated alcohol products (Scheme 49).

**Scheme 50:**
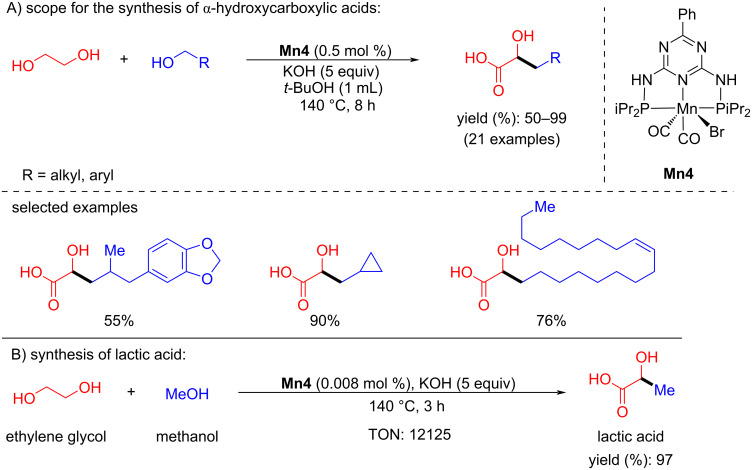
Dehydrogenative coupling of ethylene glycol and primary alcohols catalyzed by **Mn4**.

Recently, Maji’s group showed environmentally benign examples of the manganese-catalyzed dehydrogenative coupling of ethylene glycol and primary alcohols producing value-added α-hydroxycarboxylic acid molecules [77]. Several alcohols, including long-chain aliphatic alcohols, were coupled with ethylene glycol using manganese-pincer complex **Mn4** (0.5 mol %), KOH (5 equiv) in *t*-BuOH at 140 °C for 8 h under argon. Excitingly, lactic acid synthesized by treating methanol with ethylene glycol provided a very high TON of 12125. A mechanistic investigation showed the possible characteristic ^31^P NMR signals of the amido (126.5 ppm) and alkoxy (at 132.7 and 134.9 ppm) manganese complexes (Scheme 50).

#### C–C Bond formation via alkylation of esters and amides with alcohols

**Scheme 51:**
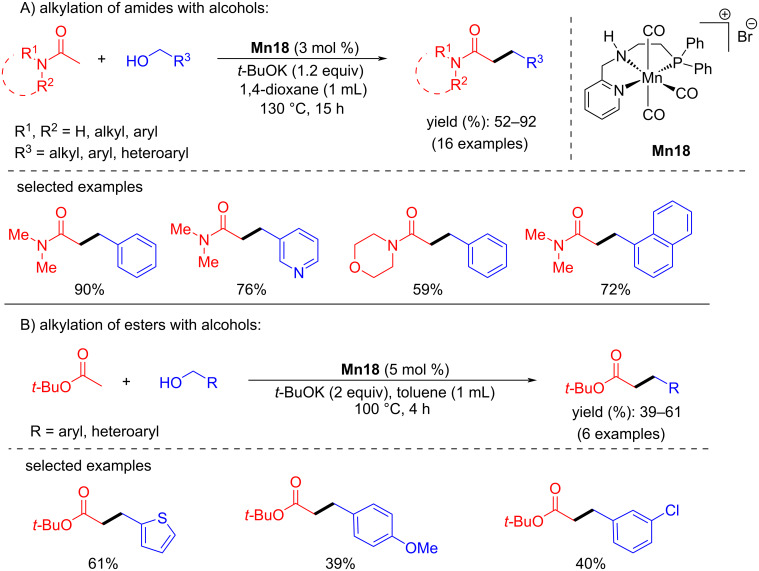
**Mn18**-cataylzed C-alkylation of unactivated esters and amides with alcohols.

Rueping and El-Sepelgy described an exciting protocol for the C-alkylation of unactivated esters and amides with alcohols using a PNN-Mn complex [78]. The alkylation of several amides with aliphatic, benzylic, and heteroaromatic alcohols gave good to excellent yields (52–92%) using **Mn18** (3 mol %) and *t*-BuOK (1.2 equiv) at 130 °C for 15 h. In the same way, alkylation of *tert*-butyl acetate with numerous alcohols was also tested using catalyst **Mn18** (5 mol %), *t*-BuOK (2 equiv) in toluene at 100 °C for 4 h and gave moderate yields of 39 to 61%. Compared to the alkylation of amides, the alkylation of esters required higher catalyst and base loading. However, the reaction proceeded at a low temperature and less reaction time (Scheme 51).

**Scheme 52:**
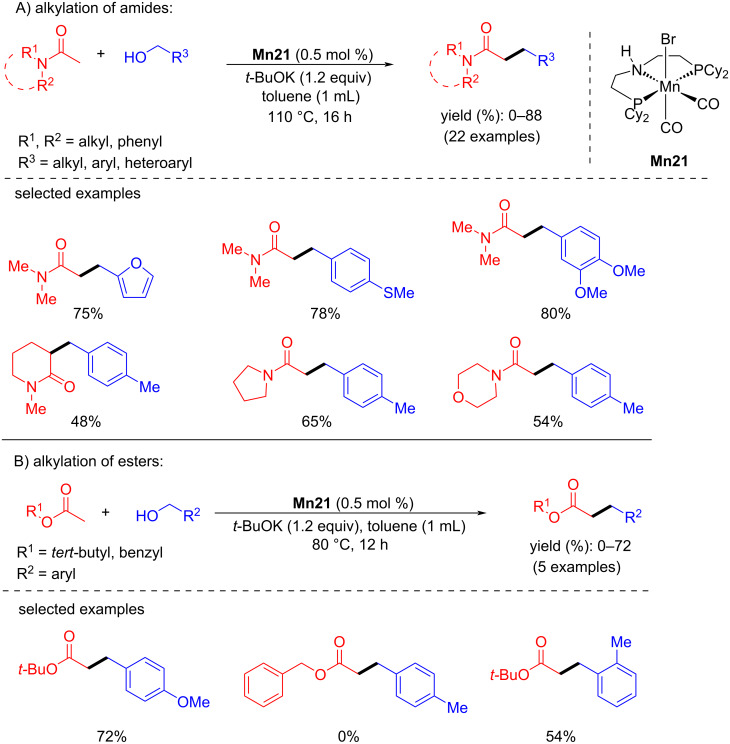
Alkylation of amides and esters using **Mn21**.

In addition, Balaraman and co-workers reported the C-alkylation of unactivated amides and *tert*-butyl acetate using primary alcohols as alkylating agents catalyzed by an aliphatic PNP-Mn pincer catalyst [79]. Various alcohols, including aliphatic alcohols, were coupled with *N*,*N*-dimethylacetamide using low catalyst loading (0.5 mol % **Mn21**) and *t*-BuOK as base (1.2 equiv) at 110 °C for 16 h and furnished the products in good yields (up to 88%). The alkylation of *tert*-butyl acetate with alcohols under the same reaction conditions provided the alkylated products with up to 72% yield at 80 °C (Scheme 52).

#### C-C Bond formation via alkylation of nitriles with alcohols

**Scheme 53:**
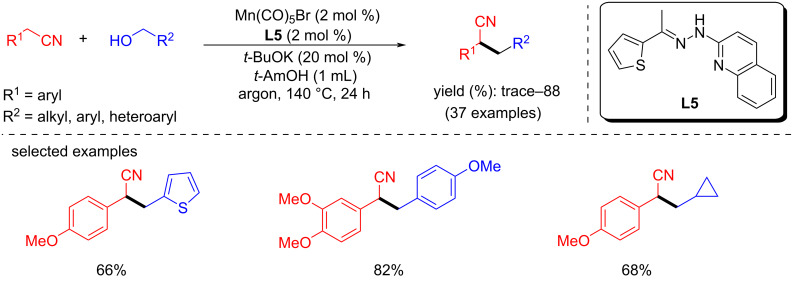
α-Alkylation of nitriles with primary alcohols using in situ-generated manganese catalyst.

In 2018, an in situ-generated manganese catalytic system for the α-alkylation of nitriles using primary alcohols was studied [80]. Various substituted nitriles were selectively alkylated in the α-position with 4-methoxybenzyl alcohol as alkyl source using Mn(CO)_5_Br (2 mol %), *t*-BuOK (20 mol %) as base in *t*-AmOH as solvent at 140 °C for 24 h to afford the products with up to 88% yield. Furthermore, several benzylic and aliphatic alcohols were used as an alkylating agent (Scheme 53).

**Scheme 54:**
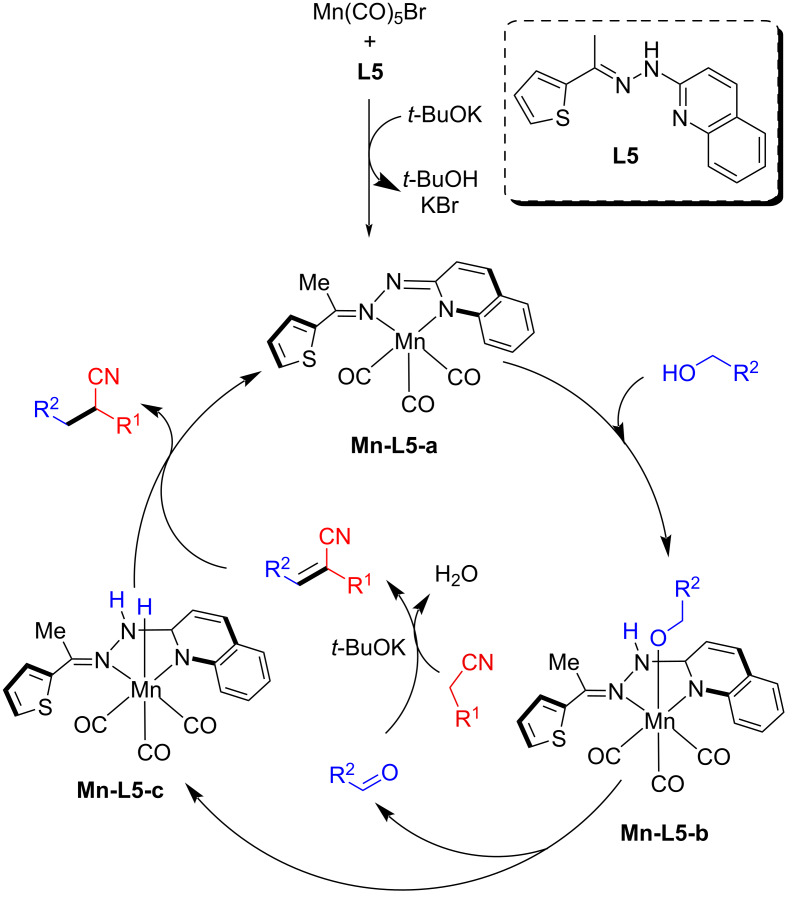
Proposed mechanism for the α-alkylation of nitriles with primary alcohols.

The proposed mechanism suggested that the dehydrogenation of the alcohol took place first using the active amido manganese complex. The formed carbonyl compound then condensed with the alkylnitrile and generated the unsaturated compound in the presence of a base. In the final step, the hydrogenation of the formed intermediate took place via the outer sphere mechanism to deliver the desired alkylated nitrile products (Scheme 54).

**Scheme 55:**
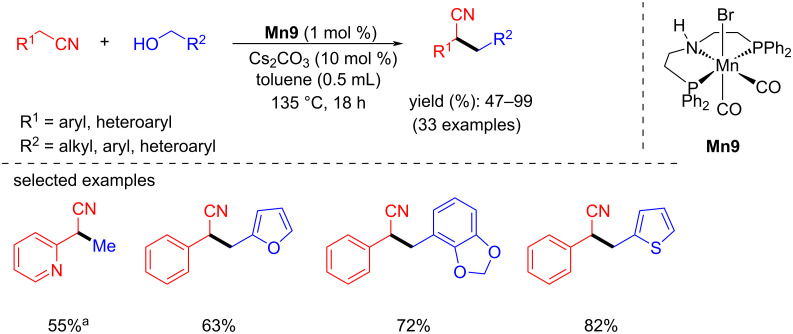
**Mn9**-catalyzed α-alkylation of nitriles with primary alcohols. ^a^1,4-Dioxane was used as solvent, 24 h.

In 2019, Rueping and El-Seplegy reported the well-defined manganese-PNP complex-catalyzed alkylation of nitriles with alcohols as a hydrogen donor [81]. Three different manganese catalysts were screened for the alkylation of phenylacetonitrile in the presence of a base. Several alcohols and nitriles were tested and showed better functional tolerance with up to 99% yield under the optimized conditions (1 mol % of **Mn9** and 10 mol % of Cs_2_CO_3_ in toluene at 135 °C for 18 h) (Scheme 55). The mechanistic investigation discussed the formation of a manganese imido complex by treating **Mn9** with 1 equiv of *t*-BuOK at room temperature (rt) for 1 h in C_6_D_6_. The signal at 91.02 ppm in the ^31^P NMR spectrum confirmed the formation of the imido compound.

#### C-Alkylation of heterocyclic compounds with alcohols

**Figure 4:**
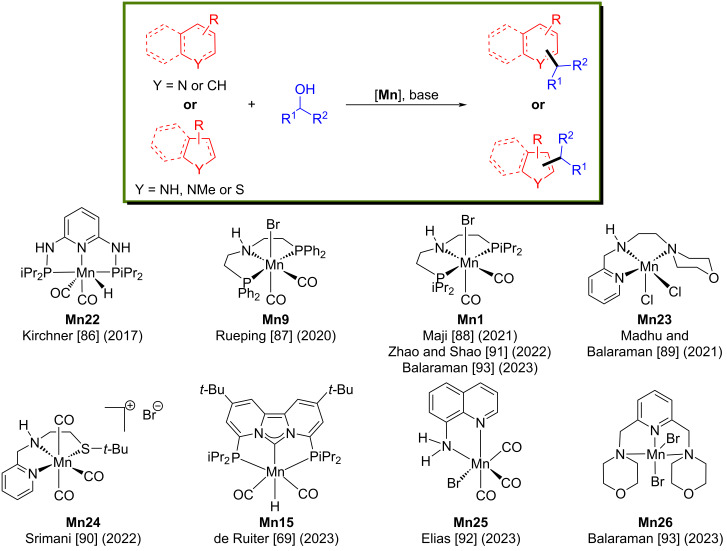
Manganese complexes used for alkylation of heterocyclic compounds.

Functionalized heterocyclic compounds are omnipresent structural skeletons in bioactive compounds [82]. Remarkably, the alkylation of indoles and quinolines received significant interest since they are common compounds in pharmaceutical and agrochemical industries [83–85]. Various manganese catalysts have been reported (Figure 4) for the C-alkylation of heterocyclic compounds with several alcohols, including indoles and quinolines.

**Scheme 56:**
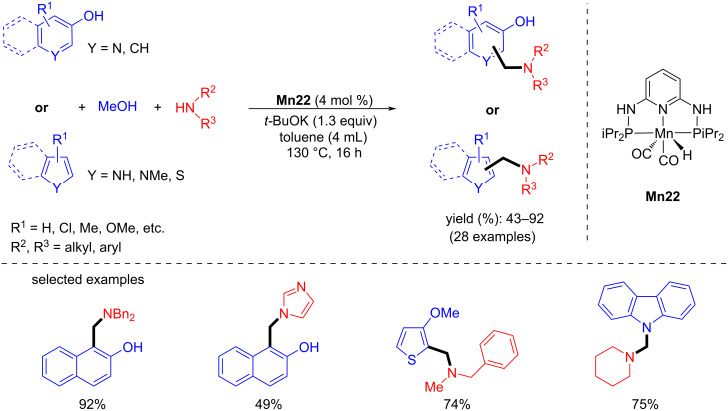
Aminomethylation of aromatic compounds with secondary amines and methanol catalyzed by **Mn22**.

In 2017, Kirchner’s group established a new method for the aminomethylation of aromatic compounds with secondary amines and methanol as a C1 source [86]. A total of 28 desired aminomethylated aromatic products were isolated with moderate to good yields (Scheme 56).

**Scheme 57:**
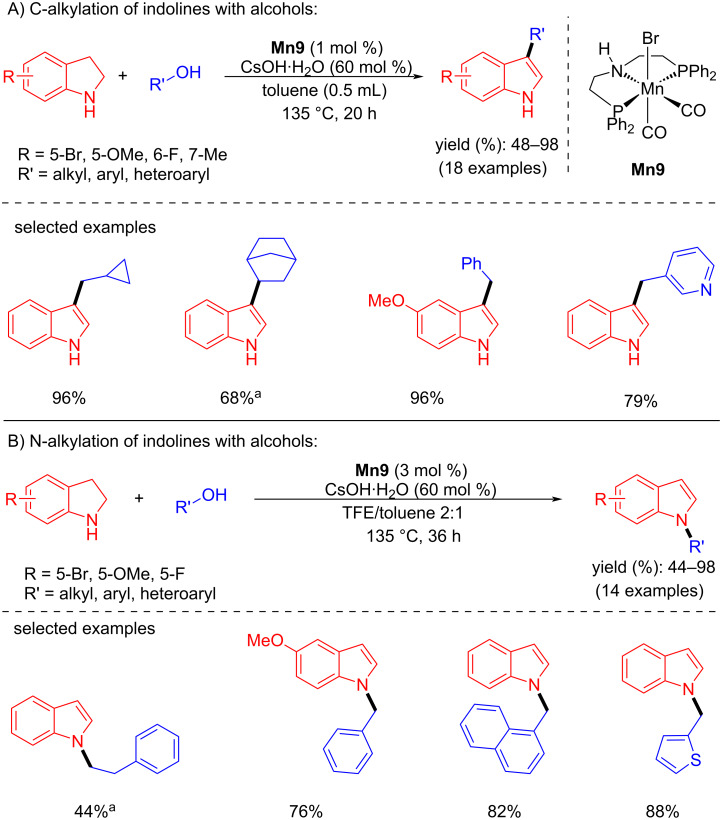
Regioselective alkylation of indolines with alcohols catalyzed by **Mn9**. ^a^**Mn9** (4 mol %), 48 h.

Later, Rueping and co-workers developed the regioselective alkylation of indolines with alcohols as the alkylating agent using a manganese-pincer catalyst [87]. Interestingly, the **Mn9**-catalyzed dehydrogenation of alcohols and indolines provided selectively the C3- or N-alkylated products depending on the solvent. For example, the alkylation of indolines with several alcohols using 1 mol % of **Mn9**, 60 mol % of CsOH·H_2_O in toluene as solvent at 135 °C for 20 h afforded the C3-alkylated indole products in up to 98% yield (Scheme 57). Similarly, the alkylation of indolines with various alcohols using 3 mol % of **Mn9** in a TFE/toluene 2:1 mixture provided the corresponding N-alkylated products.

**Scheme 58:**
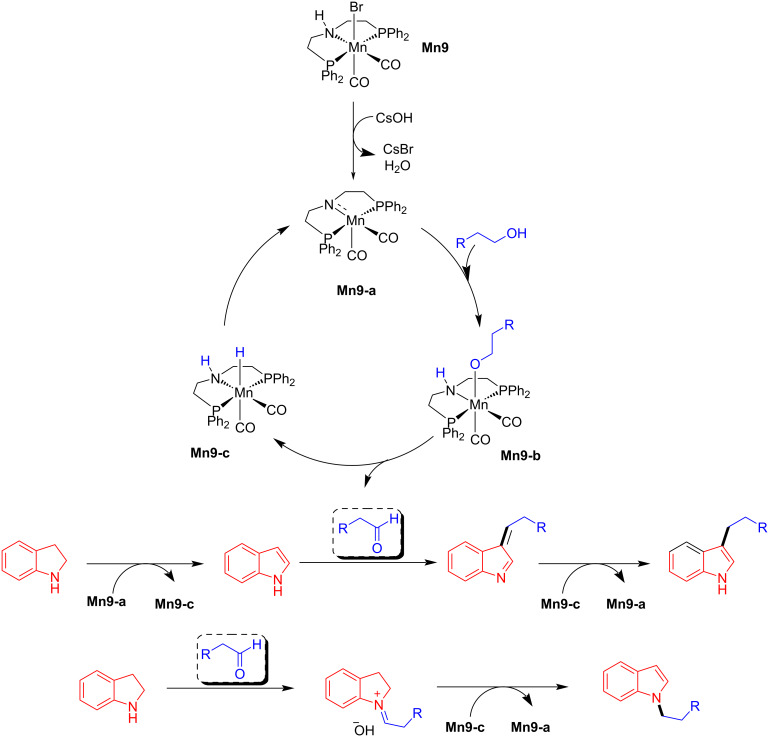
Proposed mechanism for the C- and N-alkylation of indolines with alcohols.

The mechanistic investigation showed that the metal complex activated by the base dehydrogenates the alcohol to the aldehyde and indoline to indole by acceptorless dehydrogenation. Moreover, C3 alkylation proceeded via BH (Scheme 58).

**Scheme 59:**
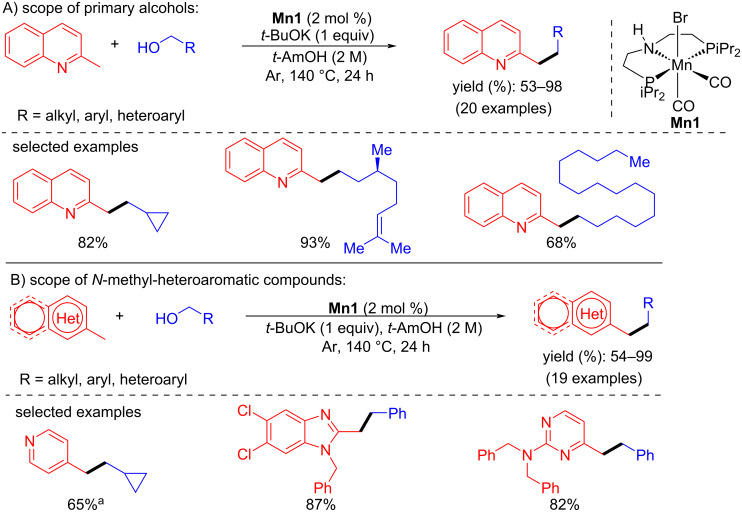
C-Alkylation of methyl *N*-heteroarenes with primary alcohols catalyzed by **Mn1**. ^a^Time was 60 h.

In early 2021, Maji’s group demonstrated an efficient approach for the C-alkylation of methyl *N*-heteroarenes with primary alcohols using a manganese-pincer complex [88]. Various methyl-substituted N-heteroarenes were coupled with several alcohols using **Mn1** (2 mol %), *t*-BuOK (1 equiv) as a base in *t*-AmOH at 140 °C under argon atmosphere for 24 h to give moderate to excellent yields (53–98%) of the desired C-alkylated N-heteroarene products (Scheme 59).

**Scheme 60:**
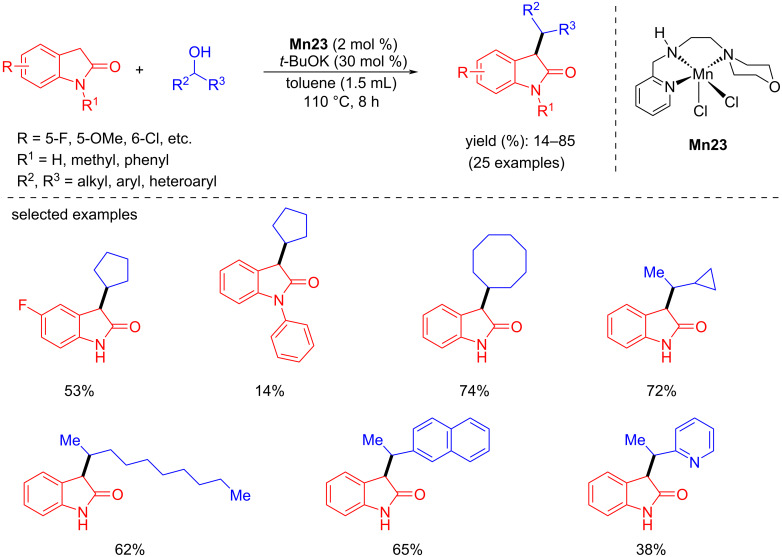
C-Alkylation of oxindoles with secondary alcohols.

The same year, Balaraman and co-workers reported the selective C-alkylation of oxindole with unactivated secondary alcohols catalyzed by a phosphine-free NNN-Mn(II) catalyst [89]. Various cyclic and aliphatic secondary alcohols were coupled with different oxindoles using 2 mol % of **Mn23** and *t*-BuOK (30 mol %) in toluene at 110 °C for 8 h to afford the C3-alkylated oxindoles with up to 85% yield (Scheme 60). However, secondary alcohols substituted with reducible (nitro, amide, aldehyde) groups and −OH, −SH, and –NHMe groups did not provide any expected C-alkylated product.

**Scheme 61:**
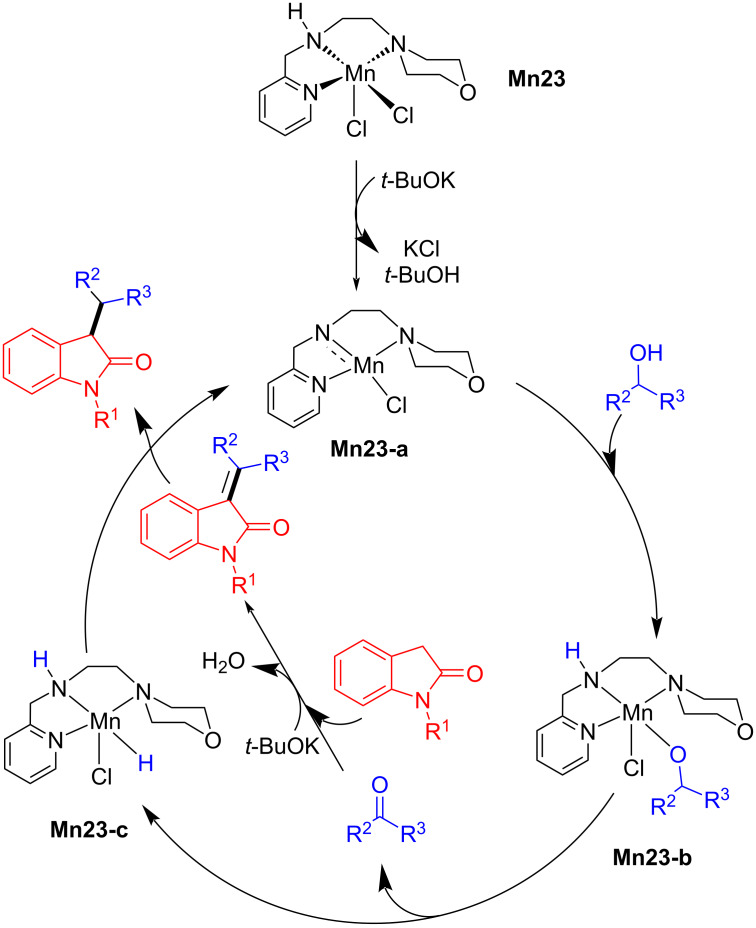
Plausible mechanism for the **Mn23**-catalyzed C-alkylation of oxindoles with secondary alcohols.

Like the previous C–C bond forming mechanism, the base-assisted aldol condensation of the ketone with the oxindole generated the unsaturated C-alkylated intermediate and H_2_O. Finally, the unsaturated product is hydrogenated to the saturated C-alkylated product by the manganese hydride complex **Mn23-c** with regeneration of the active catalyst **Mn23-a** (Scheme 61).

**Scheme 62:**
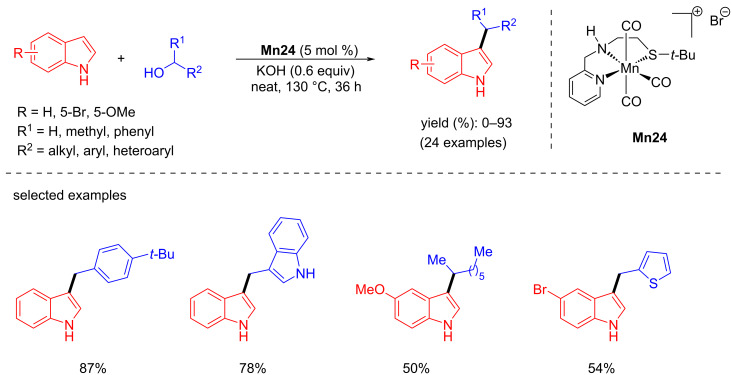
Synthesis of C-3-alkylated products by coupling alcohols with indoles and aminoalcohols.

Later, Srimani and co-workers studied the C-3 alkylation of indoles with various primary and secondary alcohols using **Mn24** (5 mol %) and KOH (0.6 equiv) under neat conditions for 36 h at 130 °C (Scheme 62) [90]. The same cationic complex was used for the synthesis of bis(indolyl)methane by coupling the same substrate using *t*-BuOK (50 mol %) as the base in toluene.

**Scheme 63:**
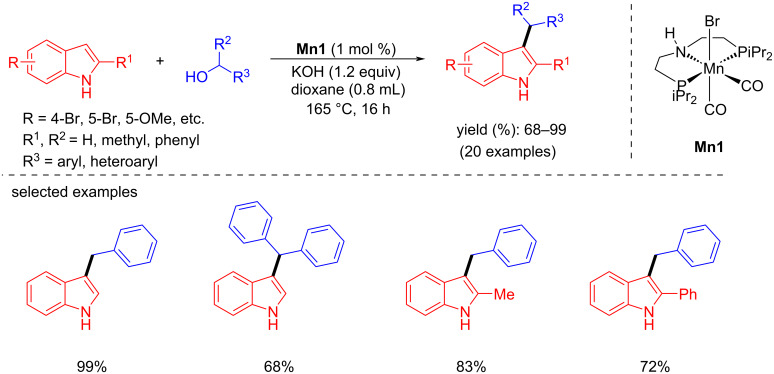
C3-Alkylation of indoles using **Mn1**.

In 2022, Shao and co-workers reported the C-3 alkylation of indoles with primary and secondary benzylic alcohols using an iPrPNP pincer-manganese complex. Three different pincer-Mn complexes were investigated for the alkylation of indole with benzyl alcohol. Among them, **Mn1** demonstrated an excellent activity with 1 mol % of **Mn1**, 1.2 equiv of KOH as base in dioxane at 165 °C for 16 h giving a 99% yield [91]. Further investigation of indole with primary benzyl alcohols under the same conditions gave up to 93% yield of the desired products (Scheme 63), whereas a secondary benzylic alcohol gave the product in 68% yield. When substituted indoles were reacted with benzyl alcohol the C-3-benzylated indole products were obtained with up to 96% yield under the same reaction conditions.

**Scheme 64:**
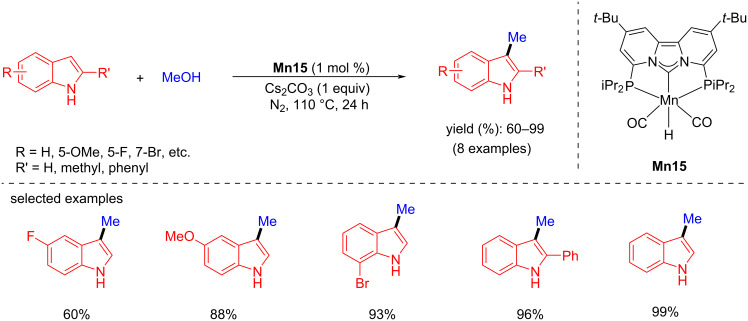
C-Methylation of indoles with **Mn15** and methanol.

After the successful attempt of using PCNHCP-based manganese complexes for the α-methylation of ketones with methanol as a C1 source by de Ruiter et al., the methylation of indole was also studied [69]. Methylation of substituted indoles with methanol was achieved using 1 mol % of **Mn15** with Cs_2_CO_3_ (1 equiv) as a base in methanol at 110 °C for 24 h under a N_2_ atmosphere, giving the desired products with 60 to 99% yields (Scheme 64).

**Scheme 65:**
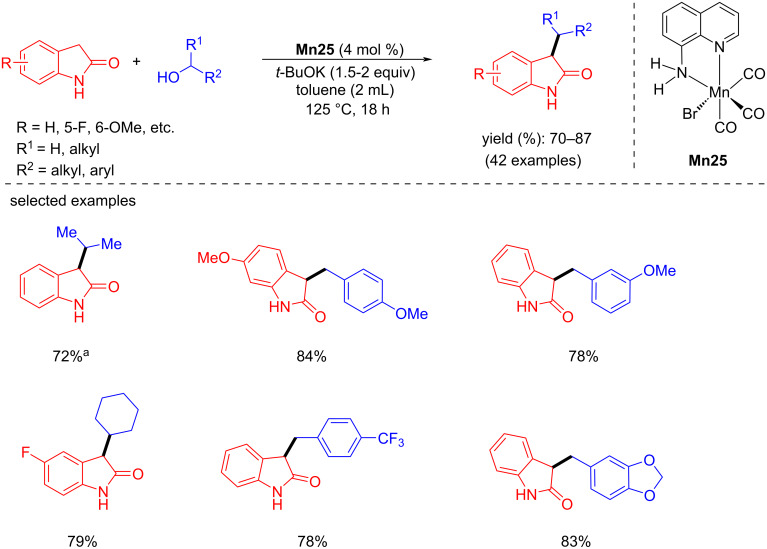
α-Alkylation of 2-oxindoles with primary and secondary alcohols catalyzed by **Mn25**. ^a^Reaction carried out without solvent.

In 2023, Elias et al. reported that an air-stable, phosphine-free manganese complex generated from 8-quinoline could α-alkylate 2-oxindole with primary and secondary alcohols [92]. Various secondary alcohols were tested with oxindoles using 4 mol % of **Mn25** as a pre-catalyst, *t*-BuOK (1.5–2 equiv.) as a base in toluene at 125 °C for 18 h to provide the C-3-alkylated oxindoles with good yields (70–87%). Moreover, substituting oxindole with different primary alcohols gave up to 85% yield of the isolated products (Scheme 65).

**Scheme 66:**
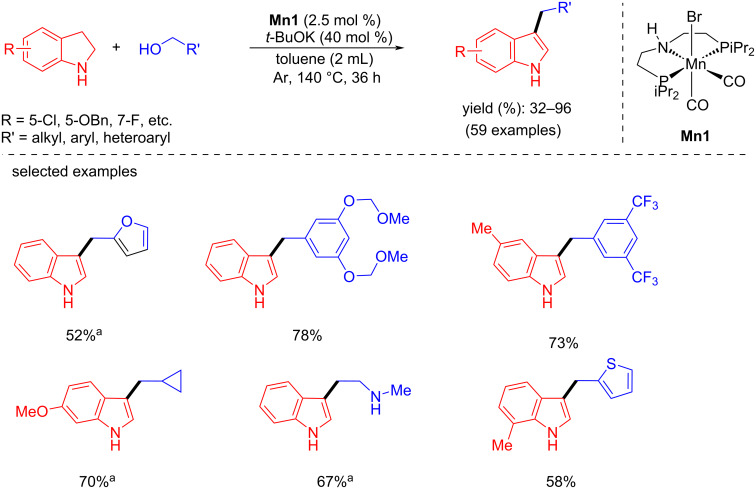
Dehydrogenative alkylation of indolines with **Mn1**. ^a^**Mn1** (5.0 mol %) was used.

Balaraman and co-workers established an innovative protocol for achieving manganese-catalyzed divergence in the C3-alkylation of indoles with alcohols. Various functionalized (hetero)aromatic and aliphatic alcohols were used as an alkylating agent for the double dehydrogenative alkylation of indolines with manganese-pincer complex **Mn1** (2.5 mol %) and *t*-BuOK (40 mol %) in toluene at 140 °C for 36 h under an argon atmosphere (Scheme 66) [93].

**Scheme 67:**
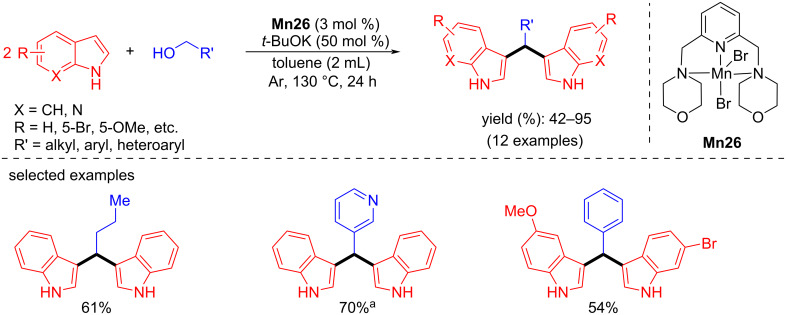
Synthesis of bis(indolyl)methane derivatives from indoles and alcohols catalyzed by **Mn26**. ^a^**Mn26** (5.0 mol %) was used.

On the other hand, numerous bis(indolyl)methane derivatives were synthesized from indoles and alcohols via an interrupted BH approach using **Mn26** (3 mol %) and *t*-BuOK (50 mol %) in toluene at 130 °C for 24 h (Scheme 67). Fascinatingly, this process was used to synthesize pharmacologically active compounds like vibrindole A and turbomycin B alkaloids and natural products like gramine and dipterine analogues. Discrete control studies with Hg and TEMPO indicated that the reactions were homogeneous and did not proceed through a radical pathway.

### Synthesis of heterocycles via C–C and C–N bond formation

**Scheme 68:**
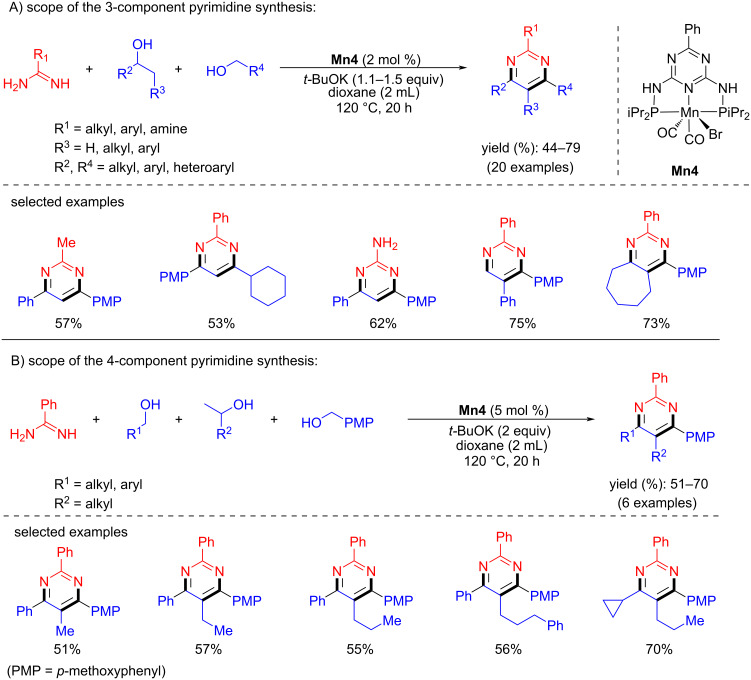
One-pot synthesis of pyrimidines via BH.

In 2016, Beller and co-workers reported an intramolecular cyclization using 2-(2-aminophenyl)ethanol for the synthesis of the corresponding indole (98% yield) at 100 °C for 48 h [34]. One year later, Kempe and co-workers showed the multicomponent synthesis of pyrimidines from amidines and alcohols using **Mn4** via C–C and C–N bond formations [94]. Various amidines were selectively coupled with different alcohols using 2 mol % of **Mn4** and 1.1–1.5 equiv of *t*-BuOK in 1,4-dioxane at 120 °C for 20 h, affording good to excellent yields of the substituted pyrimidines (Scheme 68).

**Scheme 69:**
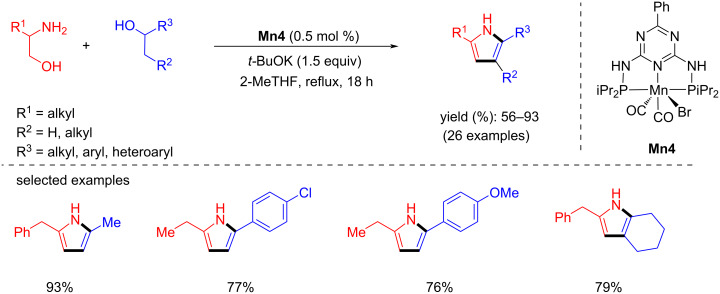
Synthesis of pyrroles from alcohols and aminoalcohols using **Mn4**.

The same group disclosed an efficient synthesis of substituted pyrroles from aminoalcohols and secondary alcohols using **Mn4** under mild conditions [95]. A variety of amino alcohols and alcohols were investigated with **Mn4** (0.5 mol %) and *t*-BuOK (1.5 equiv) in 2-MeTHF under reflux conditions and the corresponding pyrroles were isolated with up to 93% yield (Scheme 69). Notably, the same pincer ligand-supported Co and Fe complexes showed no activity in pyrrole synthesis under the same reaction conditions.

**Scheme 70:**
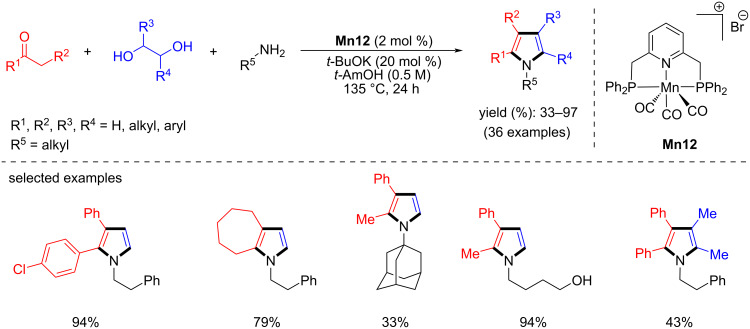
Synthesis of pyrroles via multicomponent reaction catalyzed by **Mn12**.

In 2019, Rueping, El-Sepelgy and co-workers achieved the sustainable multicomponent synthesis of pyrroles from readily available substrates catalyzed by manganese-pincer complex **Mn12** [96]. The use of 2 mol % of **Mn12** in combination with a catalytic amount of *t*-BuOK (20 mol %) in *t*-AmOH at 135 °C for 24 h allowed for the investigation of several alkyl and aryl ketones with amines and vicinal diols yielding good to excellent yields of the desired pyrroles (Scheme 70) with water and hydrogen gas being the only byproducts. DFT calculations suggested that the metal–ligand cooperation plays a crucial role in the acceptorless dehydrogenation of ethylene glycol to glycolaldehyde and the HA process.

**Scheme 71:**
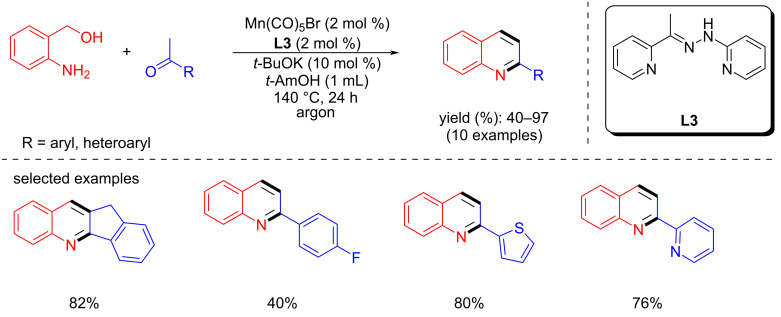
Friedländer quinoline synthesis using an in situ-generated phosphine-free manganese catalyst.

In 2018, Maji’s group reported the Friedländer quinoline synthesis using a phosphine-free manganese catalyst generated in situ from Mn(CO)_5_Br and **L3** [58]. Under optimized conditions (2 mol % Mn(CO)_5_Br, 10 mol % *t*-BuOK, *t*-AmOH, argon atmosphere), various quinoline derivatives were successfully synthesized with this protocol (Scheme 71).

**Scheme 72:**
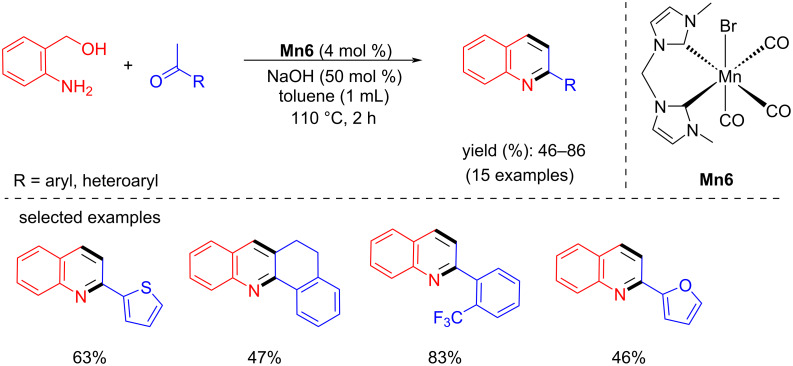
Quinoline synthesis using bis-N-heterocyclic carbene-manganese catalyst **Mn6**.

In 2019, Liu et al. used a bis-N-heterocyclic carbene-supported manganese complex for quinoline synthesis by coupling aminobenzyl alcohols and methyl ketones with **Mn6** (4 mol %), NaOH (50 mol %) in toluene at 110 °C for 2 h [64]. Moderate to good yields (46–86%) of the 2-substituted quinoline derivatives were isolated (Scheme 72).

**Scheme 73:**
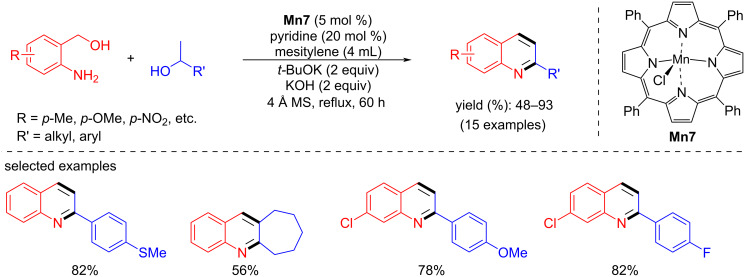
Quinoline synthesis using manganese(III)-porphyrin catalyst **Mn7**.

In the same year, Madsen’s team introduced a manganese(III)-porphyrin catalyst for the synthesis of quinoline derivatives from 2-aminobenzyl alcohols and secondary alcohols in the combination of KOH/*t*-BuOK 1:1 with 5 mol % **Mn7** catalyst loading [43]. However, a high temperature (reflux with mesitylene) and long reaction time (60 h) were required to achieve a moderate to high yields (48–93%) (Scheme 73).

**Scheme 74:**
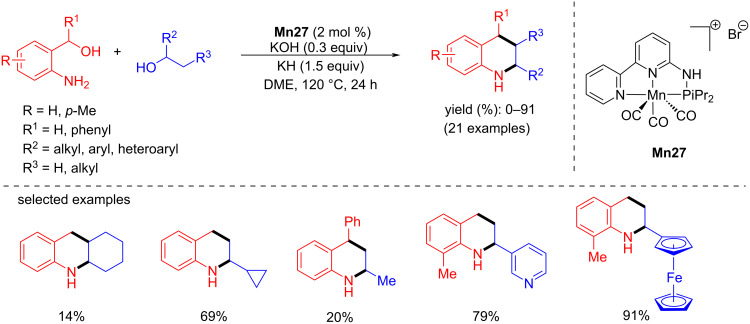
Manganese-catalyzed tetrahydroquinoline synthesis via borrowing BH.

**Scheme 75:**
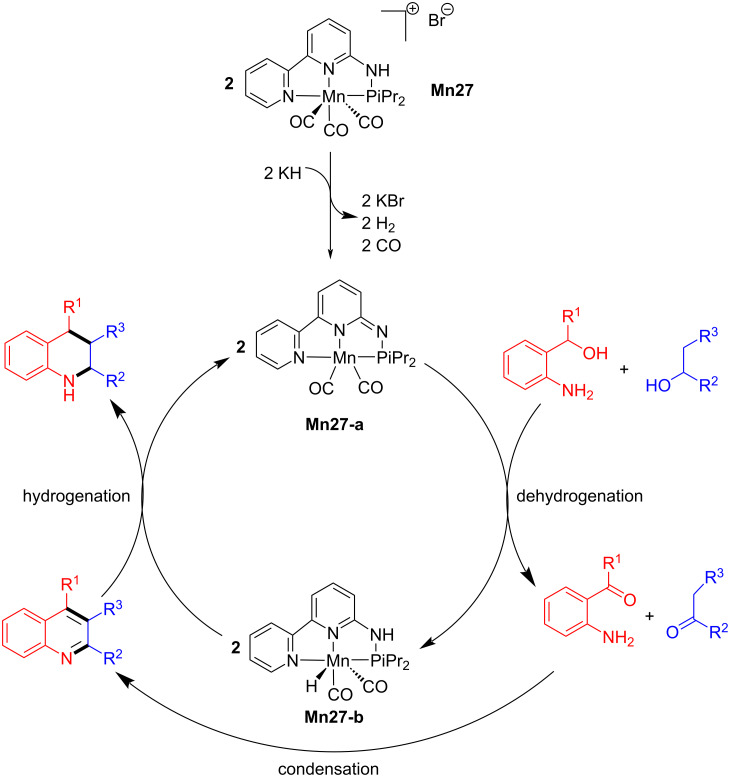
Proposed mechanism for the manganese-catalyzed tetrahydroquinoline synthesis.

Later, Hultzsch and co-workers reported a PN3 pincer-supported manganese complex for the synthesis of tetrahydroquinolines via BH methodology [97]. The reaction conditions were optimized with 2-aminobenzyl alcohol and 1-phenylethanol using **Mn27**. Among the several bases tested in DME at 120 °C for 24 h, a mixture of KH and KOH (1:5) together with 2 mol % of Mn complex **Mn27** afforded 2-phenyl-1,2,3,4-tetrahydroquinoline as product with 78% yield. Several aromatic and aliphatic substituted alcohols were studied, and the results showed good to exceptional yields of the substituted tetrahydroquinolines (Scheme 74). The active amido complex **Mn27-a** dehydrogenated the amino alcohol and secondary alcohol into the corresponding carbonyl compounds, and the subsequent base-assisted condensation allowed the formation of the quinoline product, which was further hydrogenated into the target compound (Scheme 75).

**Scheme 76:**
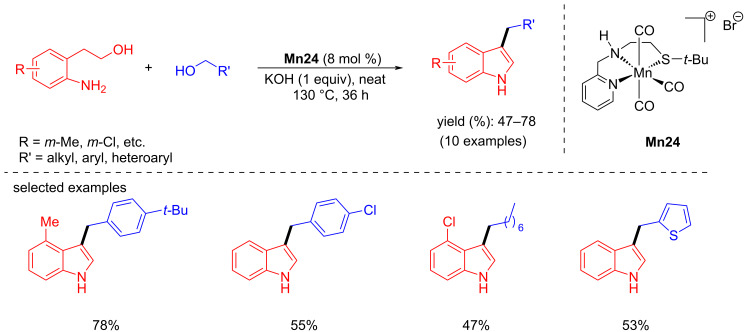
Synthesis of C3-alkylated indoles using **Mn24**.

In 2022, Srimani and co-workers showed the synthesis of C3-functionalized indoles by coupling of (2-aminophenyl)ethanol with various alcohols, including aliphatic alcohols, using **Mn24** (8 mol %) and KOH (1 equiv) under neat conditions for 36 h at 130 °C and afforded yields up to 78% (Scheme 76) [90].

**Scheme 77:**
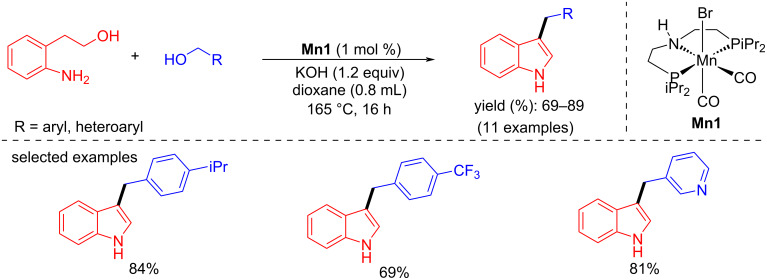
Synthesis of C-3-alkylated indoles using **Mn1**.

In 2022, Shao and co-workers synthesized C3-alkylated indoles by coupling 2-aminophenethanol with several substituted benzyl alcohols, including heteroaromatic alcohols, in good to excellent yield (69–89%) using 1 mol % of **Mn1**, 1.2 equiv of KOH as base in dioxane at 165 °C for 16 h (Scheme 77) [91].

### Miscellaneous C-alkylation reactions

**Scheme 78:**
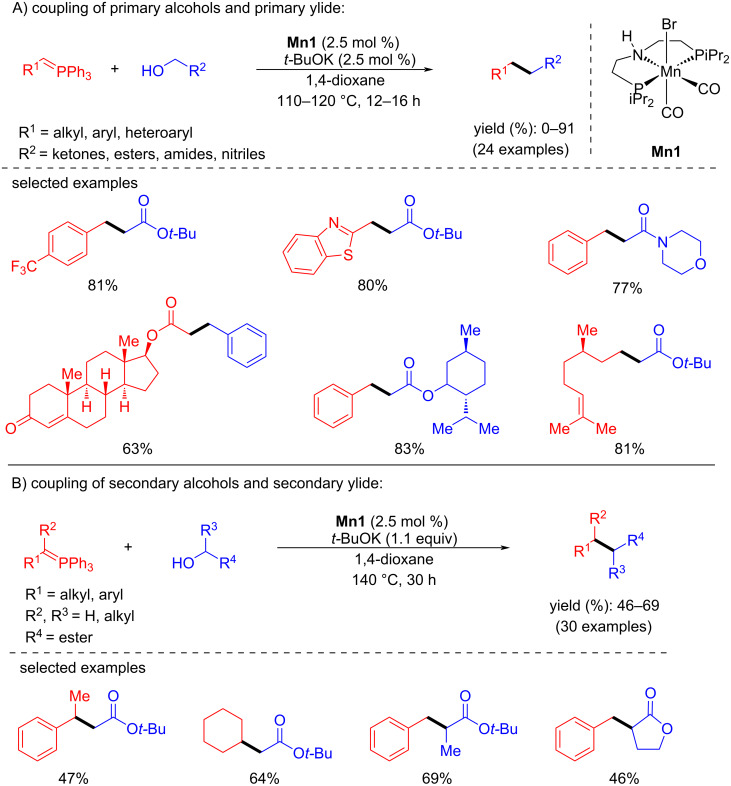
C–C Bond formation by coupling of alcohols and ylides.

In 2020, Werner used a Mn-PNP pincer catalyst for the coupling of alcohols and ylides to build C–C and C=C bonds via BH and dehydrogenative coupling (not shown), respectively [98]. Using catalyst **Mn1** and *t*-BuOK (1:1) in 1,4-dioxane at 110–120 °C for 12–16 h, some substituted benzylic alcohols and ylides were screened to produce the desired products with a yield of up to 91% (Scheme 78). However, a high temperature (140 °C), a prolonged reaction time (30 h), and an excess of base (1.1 equiv of *t*-BuOK) were needed for the coupling of secondary alcohols with secondary ylides. The observed moderate yields (46–69%) of the desired products were due to the formation of dehydrogenative side products.

**Scheme 79:**
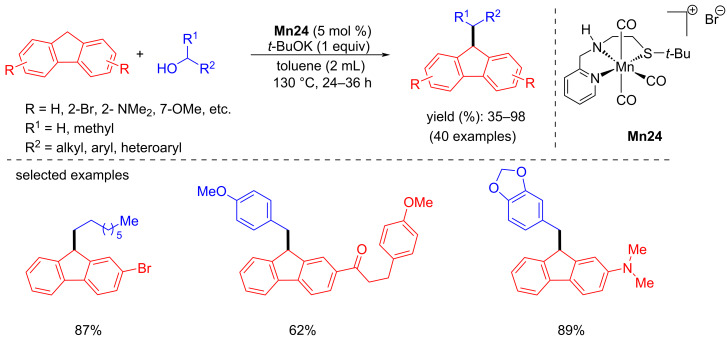
C-Alkylation of fluorene with alcohols catalyzed by **Mn24**.

**Scheme 80:**
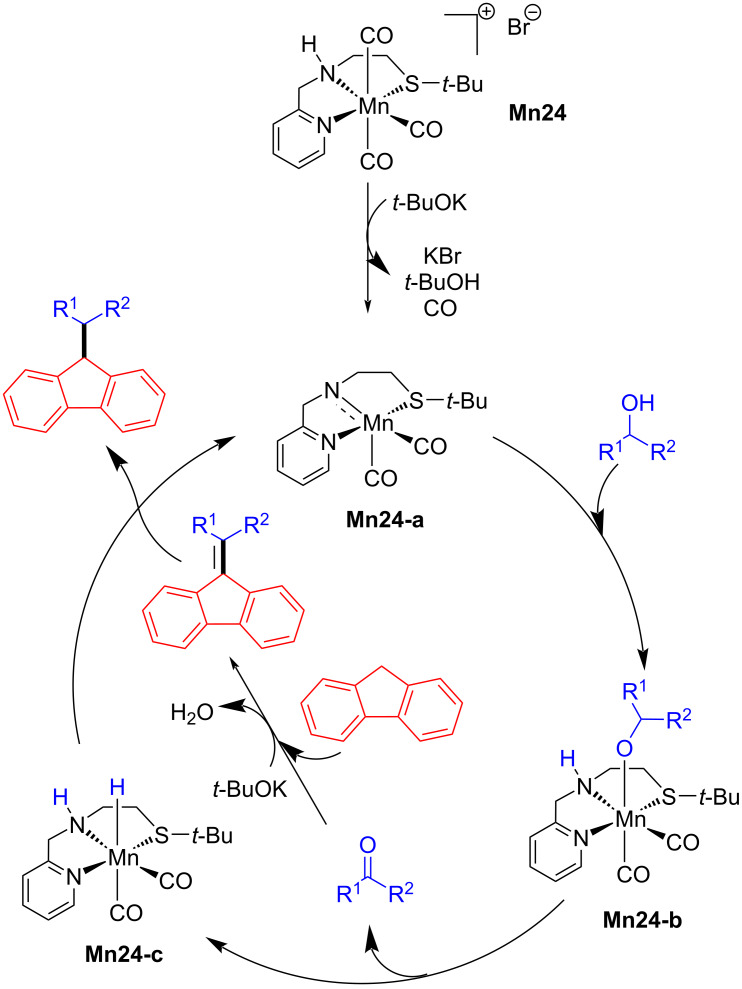
Proposed mechanism for the C-alkylation of fluorene with alcohols catalyzed by **Mn24**.

In 2021, Srimani and co-workers reported the C-alkylation and olefination (not shown) of fluorene and indene with alcohols using phosphine-free and air-stable Mn-NNS complexes [99]. Various types of alcohols, including aliphatic and secondary alcohols, were coupled with fluorene and indene, giving good to excellent yields (35–98%) of the desired alkylated products using **Mn24** (5 mol %), *t*-BuOK (1 equiv) in toluene at 130 °C for 24–36 h (Scheme 79). A similar C–C bond-formation mechanism was proposed in earlier reports. Fluorene coupled with the carbonyl compound in the presence of base led to the unsaturated compound, which was hydrogenated by **Mn24-c** to deliver the desired final product (Scheme 80).

**Scheme 81:**
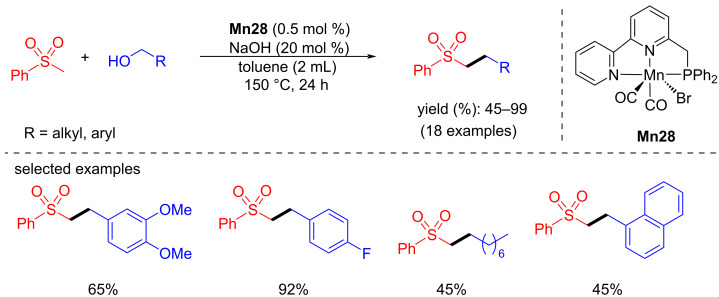
α-Alkylation of sulfones using Mn-PNN catalyst **Mn28**.

In 2023, Milstein et al. reported the α-alkylation of sulfones catalyzed by a Mn-PNN catalyst using alcohols as an alkylating agent [100]. The reaction conditions were optimized with benzyl alcohols and methyl phenyl sulfones using four different catalysts. Among all, 0.5 mol % of **Mn28** and 20 mol % of NaOH in toluene at high temperature (150 °C) for 24 h gave better selectivity and yields towards the desired alkylated products. Under the optimized conditions, several alcohols were investigated and gave the products in isolated yields up to 99% (Scheme 81).

## Conclusion

Manganese-catalyzed borrowing hydrogen reactions have emerged as powerful tools for C–C and C–N bond formation from readily available alcohols. A series of homogeneous manganese catalyst systems have been successfully established, and good catalytic activity and selectivity have been obtained for C-alkylation and N-alkylation reactions. As evident in the multicomponent reactions, manganese is recognized as a potent catalyst to replace the expensive iridium metal in BH reactions.

Though remarkable advances have been realized in manganese catalysis, the development of new and inexpensive ligand-supported manganese catalysts and a deeper understanding of the reaction mechanisms are expected to expand the efficiency and scope of this process. Compared to the classical BH with benzylic alcohols, the use of methanol and ethanol is really challenging since it requires a higher energy for the activation. Hence, increased catalyst or base loading and elevated temperature are needed for the N-methylation of amines with methanol compared to benzylic alcohols [41,44]. Similarly, to access heterocycles by coupling of amino alcohols and primary or secondary alcohols required harsh reaction conditions and high catalyst loading [43,90]. Hence, the development of efficient catalytic systems with base-free and mild reaction conditions for various applications such as multicomponent reactions, heterocycle synthesis, polymer synthesis, and upgrading of alcohols present exciting opportunities for future research in this field. We sincerely hope this review will provide insight towards the design of the new manganese catalysts and the study of C–C and C–N bond formation via BH reaction.

## Data Availability

Data sharing is not applicable as no new data was generated or analyzed in this study.

## References

[1] Ruiz-Castillo P, Buchwald S L (2016). Chem Rev.

[2] Devendar P, Qu R-Y, Kang W-M, He B, Yang G-F (2018). J Agric Food Chem.

[3] Leonard J, Blacker A J, Marsden S P, Jones M F, Mulholland K R, Newton R (2015). Org Process Res Dev.

[4] Corma A, Navas J, Sabater M J (2018). Chem Rev.

[5] Reed-Berendt B G, Latham D E, Dambatta M B, Morrill L C (2021). ACS Cent Sci.

[6] Nixon T D, Whittlesey M K, Williams J M J (2009). Dalton Trans.

[7] Jafarzadeh M, Sobhani S H, Gajewski K, Kianmehr E (2022). Org Biomol Chem.

[8] Nallagangula M, Subaramanian M, Kumar R, Balaraman E (2023). Chem Commun.

[9] Yan Q, Wu X, Jiang H, Wang H, Xu F, Li H, Zhang H, Yang S (2024). Coord Chem Rev.

[10] Corma A, Iborra S, Velty A (2007). Chem Rev.

[11] Hamid M H S A, Slatford P A, Williams J M J (2007). Adv Synth Catal.

[12] Hameury S, Bensalem H, De Oliveira Vigier K (2022). Catalysts.

[13] Bullock R M (2013). Science.

[14] Reed-Berendt B G, Polidano K, Morrill L C (2019). Org Biomol Chem.

[15] Subaramanian M, Sivakumar G, Balaraman E (2021). Chem Rec.

[16] Elangovan S, Topf C, Fischer S, Jiao H, Spannenberg A, Baumann W, Ludwig R, Junge K, Beller M (2016). J Am Chem Soc.

[17] Mukherjee A, Nerush A, Leitus G, Shimon L J W, Ben David Y, Espinosa Jalapa N A, Milstein D (2016). J Am Chem Soc.

[18] Maji B, Barman M K (2017). Synthesis.

[19] Wang Y, Wang M, Li Y, Liu Q (2021). Chem.

[20] Das K, Waiba S, Jana A, Maji B (2022). Chem Soc Rev.

[21] Rohit K R, Radhika S, Saranya S, Anilkumar G (2020). Adv Synth Catal.

[22] Waiba S, Maji B (2020). ChemCatChem.

[23] Nad P, Mukherjee A (2021). Asian J Org Chem.

[24] Das K, Barman M K, Maji B (2021). Chem Commun.

[25] Lawrence S A (2004). Amines: Synthesis Properties, and Applications.

[26] Monnier F, Taillefer M (2009). Angew Chem, Int Ed.

[27] Bagal D B, Bhanage B M (2015). Adv Synth Catal.

[28] Heravi M M, Kheilkordi Z, Zadsirjan V, Heydari M, Malmir M (2018). J Organomet Chem.

[29] Kalck P, Urrutigoïty M (2018). Chem Rev.

[30] Afanasyev O I, Kuchuk E, Usanov D L, Chusov D (2019). Chem Rev.

[31] Guillena G, Ramón D J, Yus M (2010). Chem Rev.

[32] Bähn S, Imm S, Neubert L, Zhang M, Neumann H, Beller M (2011). ChemCatChem.

[33] Podyacheva E, Afanasyev O I, Vasilyev D V, Chusov D (2022). ACS Catal.

[34] Elangovan S, Neumann J, Sortais J-B, Junge K, Darcel C, Beller M (2016). Nat Commun.

[35] Neumann J, Elangovan S, Spannenberg A, Junge K, Beller M (2017). Chem – Eur J.

[36] Bruneau-Voisine A, Wang D, Dorcet V, Roisnel T, Darcel C, Sortais J-B (2017). J Catal.

[37] Fertig R, Irrgang T, Freitag F, Zander J, Kempe R (2018). ACS Catal.

[38] Das U K, Ben‐David Y, Diskin‐Posner Y, Milstein D (2018). Angew Chem, Int Ed.

[39] Landge V G, Mondal A, Kumar V, Nandakumar A, Balaraman E (2018). Org Biomol Chem.

[40] Reed-Berendt B G, Morrill L C (2019). J Org Chem.

[41] Huang M, Li Y, Li Y, Liu J, Shu S, Liu Y, Ke Z (2019). Chem Commun.

[42] Homberg L, Roller A, Hultzsch K C (2019). Org Lett.

[43] Azizi K, Akrami S, Madsen R (2019). Chem – Eur J.

[44] Reed‐Berendt B G, Mast N, Morrill L C (2020). Eur J Org Chem.

[45] Das K, Kumar A, Jana A, Maji B (2020). Inorg Chim Acta.

[46] Wei D, Yang P, Yu C, Zhao F, Wang Y, Peng Z (2021). J Org Chem.

[47] Babu R, Sukanya Padhy S, Kumar R, Balaraman E (2023). Chem – Eur J.

[48] Brodie C N, Owen A E, Kolb J S, Bühl M, Kumar A (2023). Angew Chem, Int Ed.

[49] Friães S, Gomes C S B, Royo B (2023). Organometallics.

[50] Miyaura N, Suzuki A (1995). Chem Rev.

[51] Zheng Y-L, Newman S G (2021). Chem Commun.

[52] Reetz M T (1982). Angew Chem, Int Ed Engl.

[53] Caine D (1991). Alkylations of Enols and Enolates. Comprehensive Organic Synthesis.

[54] Zhao F, Tan B, Li Q, Tan Q, Huang H (2022). Molecules.

[55] Huang F, Liu Z, Yu Z (2016). Angew Chem, Int Ed.

[56] Yang D-Y, Wang H, Chang C-R (2022). Adv Synth Catal.

[57] Peña‐López M, Piehl P, Elangovan S, Neumann H, Beller M (2016). Angew Chem, Int Ed.

[58] Barman M K, Jana A, Maji B (2018). Adv Synth Catal.

[59] Chakraborty S, Daw P, Ben David Y, Milstein D (2018). ACS Catal.

[60] Kabadwal L M, Das J, Banerjee D (2018). Chem Commun.

[61] Sklyaruk J, Borghs J C, El‐Sepelgy O, Rueping M (2019). Angew Chem, Int Ed.

[62] Bruneau-Voisine A, Pallova L, Bastin S, César V, Sortais J-B (2019). Chem Commun.

[63] Gawali S S, Pandia B K, Pal S, Gunanathan C (2019). ACS Omega.

[64] Lan X-B, Ye Z, Huang M, Liu J, Liu Y, Ke Z (2019). Org Lett.

[65] Kaithal A, Gracia L-L, Camp C, Quadrelli E A, Leitner W (2019). J Am Chem Soc.

[66] Jana A, Das K, Kundu A, Thorve P R, Adhikari D, Maji B (2020). ACS Catal.

[67] Waiba S, Jana S K, Jati A, Jana A, Maji B (2020). Chem Commun.

[68] Patra K, Laskar R A, Nath A, Bera J K (2022). Organometallics.

[69] Thenarukandiyil R, Kamte R, Garhwal S, Effnert P, Fridman N, de Ruiter G (2023). Organometallics.

[70] Jalwal S, Regina A, Atreya V, Paranjothy M, Chakraborty S (2024). Dalton Trans.

[71] Liu T, Wang L, Wu K, Yu Z (2018). ACS Catal.

[72] El‐Sepelgy O, Matador E, Brzozowska A, Rueping M (2019). ChemSusChem.

[73] Liu Y, Shao Z, Wang Y, Xu L, Yu Z, Liu Q (2019). ChemSusChem.

[74] Schlagbauer M, Kallmeier F, Irrgang T, Kempe R (2020). Angew Chem, Int Ed.

[75] Lan X-B, Ye Z, Liu J, Huang M, Shao Y, Cai X, Liu Y, Ke Z (2020). ChemSusChem.

[76] Sun F, Huang J, Wei Z, Tang C, Liu W (2023). Angew Chem, Int Ed.

[77] Waiba S, Maji K, Maiti M, Maji B (2023). Angew Chem, Int Ed.

[78] Jang Y K, Krückel T, Rueping M, El-Sepelgy O (2018). Org Lett.

[79] Rana J, Gupta V, Balaraman E (2019). Dalton Trans.

[80] Jana A, Reddy C B, Maji B (2018). ACS Catal.

[81] Borghs J C, Tran M A, Sklyaruk J, Rueping M, El-Sepelgy O (2019). J Org Chem.

[82] Horton D A, Bourne G T, Smythe M L (2003). Chem Rev.

[83] Somei M, Yamada F (2004). Nat Prod Rep.

[84] Shiri M (2012). Chem Rev.

[85] Elebiju O F, Ajani O O, Oduselu G O, Ogunnupebi T A, Adebiyi E (2023). Front Chem (Lausanne, Switz).

[86] Mastalir M, Pittenauer E, Allmaier G, Kirchner K (2017). J Am Chem Soc.

[87] Borghs J C, Zubar V, Azofra L M, Sklyaruk J, Rueping M (2020). Org Lett.

[88] Jana A, Kumar A, Maji B (2021). Chem Commun.

[89] Rana J, Nagarasu P, Subaramanian M, Mondal A, Madhu V, Balaraman E (2021). Organometallics.

[90] Mondal A, Sharma R, Dutta B, Pal D, Srimani D (2022). J Org Chem.

[91] Zhao M, Li X, Zhang X, Shao Z (2022). Chem – Asian J.

[92] Saini P, Dolui P, Nair A, Verma A, Elias A J (2023). Chem – Asian J.

[93] Mondal A, Kumar R, Suresh A K, Sahoo M K, Balaraman E (2023). Catal Sci Technol.

[94] Deibl N, Kempe R (2017). Angew Chem, Int Ed.

[95] Kallmeier F, Dudziec B, Irrgang T, Kempe R (2017). Angew Chem, Int Ed.

[96] Borghs J C, Azofra L M, Biberger T, Linnenberg O, Cavallo L, Rueping M, El‐Sepelgy O (2019). ChemSusChem.

[97] Hofmann N, Homberg L, Hultzsch K C (2020). Org Lett.

[98] Liu X, Werner T (2021). Adv Synth Catal.

[99] Mondal A, Sharma R, Pal D, Srimani D (2021). Chem Commun.

[100] Lu L, Luo J, Milstein D (2023). ACS Catal.

